# Direct Detection of Carbapenemase-Producing Klebsiella pneumoniae by MALDI-TOF Analysis of Full Spectra Applying Machine Learning

**DOI:** 10.1128/jcm.01751-22

**Published:** 2023-05-18

**Authors:** Eva Gato, Manuel J. Arroyo, Gema Méndez, Ana Candela, Bruno Kotska Rodiño-Janeiro, Javier Fernández, Belén Rodríguez-Sánchez, Luis Mancera, Jorge Arca-Suárez, Alejandro Beceiro, Germán Bou, Marina Oviaño

**Affiliations:** a Servicio de Microbiología, Complejo Hospitalario Universitario A Coruña, A Coruña, Spain; b Clover Bioanalytical Software S.L., Granada, Spain; c Servicio de Microbiología, Hospital Central de Asturias, Oviedo, Spain; d Servicio de Microbiología, Hospital General Gregorio Marañón, Madrid, Spain; e Centro de Investigación Biomedica en Red Enfermedades Infecciosas (CIBERINFEC). Instituto de Salud Carlos III (ISCIII), Madrid, Spain; NorthShore University HealthSystem

## Abstract

MALDI-TOF MS is considered to be an important tool for the future development of rapid microbiological techniques. We propose the application of MALDI-TOF MS as a dual technique for the identification of bacteria and the detection of resistance, with no extra hands-on procedures. We have developed a machine learning approach that uses the random forest algorithm for the direct prediction of carbapenemase-producing Klebsiella pneumoniae (CPK) isolates, based on the spectra of complete cells. For this purpose, we used a database of 4,547 mass spectra profiles, including 715 unduplicated clinical isolates that are represented by 324 CPK with 37 different ST. The impact of the culture medium was determinant in the CPK prediction, being that the isolates were tested and cultured in the same media, compared to the isolates used to build the model (blood agar). The proposed method has an accuracy of 97.83% for the prediction of CPK and an accuracy of 95.24% for the prediction of OXA-48 or KPC carriage. For the CPK prediction, the RF algorithm yielded a value of 1.00 for both the area under the receiver operating characteristic curve and the area under the precision-recall curve. The contribution of individual mass peaks to the CPK prediction was determined using Shapley values, which revealed that the complete proteome, rather than a series of mass peaks or potential biomarkers (as previously suggested), is responsible for the algorithm-based classification. Thus, the use of the full spectrum, as proposed here, with a pattern-matching analytical algorithm produced the best outcome. The use of MALDI-TOF MS coupled with machine learning algorithm processing enabled the identification of CPK isolates within only a few minutes, thereby reducing the time to detection of resistance.

## INTRODUCTION

The increasing emergence of carbapenemase-producing Klebsiella pneumoniae (CPK) is recognized as a global health concern by different organizations, such as the European Centre for Disease Control (ECDC), the Centers for Disease Control and Prevention (CDC), and the World Health Organization (WHO) (1–4), as infections produced by these bacteria are associated with substantial morbidity, mortality, and health care costs (5). Carbapenemases can confer resistance to almost all available beta-lactams, which are the antibiotics most commonly used to treat infections caused by Enterobacterales (6). Thus, early identification can improve the choice of therapeutic options. The detection of antimicrobial resistance is usually based on widely approved molecular techniques (7). However, these techniques are more time-consuming and expensive than matrix-assisted laser desorption/ionization time-of-flight (MALDI-TOF). In addition, molecular techniques are generally narrow-spectrum assays with single gene targets, and whole-genome sequencing (WGS) techniques are required to identify the bacterial genome. With current phenotypic and culture-based methods for the detection of antimicrobial resistance, the time from sample collection to resistance reporting can take up to 48 to 72 h, since it is necessary to isolate bacteria first, while using MALDI-TOF as a dual technique, as for the identification of bacteria and the detection of resistance, both notifications can be provided at the same time.

MALDI-TOF MS proteomics-based biotyping is used to identify microorganisms via the analysis of ribosomal proteins from whole cells ranging in size from 2 to 20 kDa, which indicates the high diversity of these proteins among different species of bacteria (8). MALDI-TOF MS can be used to characterize, in only a few minutes, the protein composition of individual bacterial species (9), and it is considered to be important in the future development of rapid microbiological techniques (10). The method is already being implemented in many clinical microbiology laboratories, unlike molecular techniques such as WGS (11). The extraction of additional information directly from the MALDI-TOF mass spectra could also enable the detection of antimicrobial resistance (12, 13). Manual methods, including washing, concentrating, and incubating microorganisms with different antibiotics, have produced good results (14, 15). However, the need to speed up the procedures and increase the traceability of the results makes the dual technique (bacterial identification and antimicrobial resistance detection) increasingly in demand in clinical laboratories.

In the field of antibiotic resistance detection, MALDI-TOF MS was initially used to detect markers associated with resistance (16–18). However, the absence of a comprehensive and reliable catalogue of markers for all potential pathogens and drug combinations led to a shift to more sophisticated approaches. Machine learning tools, such as neural networks, support vector machines, and random forests, are powerful classification systems that have been used in the health sciences, such as cancer genomics (19, 20). As advancements in high-throughput technologies generate large amounts of data, such classification features are suitable for application in proteomics-based clinical microbiological diagnoses. Several MALDI-TOF MS-based procedures and data analysis procedures have been developed. However, there remain some inconsistencies regarding the biological and technical reproducibility of these techniques. Furthermore, the absence of a universal database of reference mass spectra limits the overall applicability of MALDI-TOF MS as a first-line clinical tool (21–24).

In a previously published paper, we presented a MALDI-TOF MS data analysis pipeline for carbapenemase-producing K. pneumoniae detection using a machine learning analysis that was implemented in the online Clover MS Data Analysis Software (Clover Biosoft, Spain) (25). We have since included a larger collection of K. pneumoniae isolates to minimize the effect of different collection sites and to improve the prediction by tuning the model with a labeled data set. The aim of the present study was to validate the procedure and demonstrate that the direct tracking of CPK isolates is possible using MALDI-TOF MS in a clinical setting within 24 h of sample collection.

## MATERIALS AND METHODS

### Bacterial isolates.

The study included a representative collection of 715 unduplicated clinical isolates (Table S1). 324 of the 715 isolates were CPK. Among the 324 CPK studied, 307 were collected during a nationwide survey of carbapenemase-producing Enterobacterales (CPE) that involved 15 hospitals throughout Spain. The survey was promoted by the Spanish Society of Infectious Diseases and Clinical Microbiology (SEIMC) and by the Spanish Network for Research in Infectious Diseases (REIPI), during a two-month period in 2018. The other 17 isolates form part of our own collection. The 391 non-carbapenemase-producing K. pneumoniae (NCPK) isolates were collected in various different hospitals in Spain that were surveyed. The isolates were screened for carbapenemase production, according to the screening cutoff values recommended by the European Committee on Antimicrobial Susceptibility Testing (EUCAST) (i.e., those with a meropenem or ertapenem MIC of higher than 0.125 mg/L, as obtained via an automated microdilution method) (MicroScan [Beckman Coulter, California]) and Vitek (bioMérieux, Francia).

The 307 isolates belonging to the national survey were characterized via WGS. Total genomic DNA was obtained using a Genomic DNA Buffer Set with a Genomic-Tip 20/G (Qiagen). Purified genomic DNA from all isolates was sequenced in parallel using short-read (Illumina MiSeq benchtop, Illumina) (26) and long-read (MinION, Oxford Nanopore Technologies) approaches. The resultant long and short reads from each isolate were assembled using the Unicycler v0.4.6 hybrid assembler. The contigs were visualized using the Bandage software package (27). The assemblies that were obtained were finally annotated using Prokka v1.13 (28). The BioProject accession number for the strain genomes is PRJEB39112. An *in silico* analysis of the total antimicrobial resistance gene content of the isolates was carried out using the Resfinder v3.2 software package and the Comprehensive Antibiotic Resistance Database (CARD) (29). Multilocus sequence typing (MLSTs) was determined *in silico* from the assembled whole-genome sequencing data, using available online databases (https://cge.food.dtu.dk/services/MLST/).

The 17 isolates in our laboratory collection were characterized by routinely used genomic techniques. A PCR assay was performed to detect the genes coding for the carbapenemases OXA-48 and KPC. DNA was extracted using the boiling method. Specific oligonucleotides were used to amplify the different genes (OXA-48 Fw: GCGTGGTTAAGGATGAACAC; OXA-48 Rv: CATCAAGTTCAACCCAACCG; KPC Fw: CGTCTAGTTCTGCTGTCTTG; KPC Rv: CTTGTCATCCTTGTTAGGCG) (30). The presence of the different carbapenemase genes was confirmed by sequencing the PCR products. The MLST analysis of K. pneumoniae was conducted in accordance with the reference protocol (https://bigsdb.pasteur.fr/klebsiella/), under the following conditions: initial denaturation at 94°C for 2 min; 35 cycles of 20s at 94°C, 30 s at 50°C, and 30 s at 72°C; and final elongation for 5 min at 72°C. The nucleotide sequences were compared with existing entries in the MLST database (https://bigsdb.pasteur.fr/cgi-bin/bigsdb/bigsdb.pl?db=pubmlst_klebsiella_seqdef&page=sequenceQuery) for the generation of allelic numbers and the assignment of STs.

Isolates that did not fulfil the EUCAST criteria for carbapenemase screening were classified as non-carbapenemase-producing isolates. Those isolates were not analyzed for their antimicrobial resistance gene content or clonal complex.

The bacterial isolates were randomly divided into two sets: a training set and a validation set (Table S1).

### Acquisition of MALDI-TOF MS spectra.

**FIG 1:**
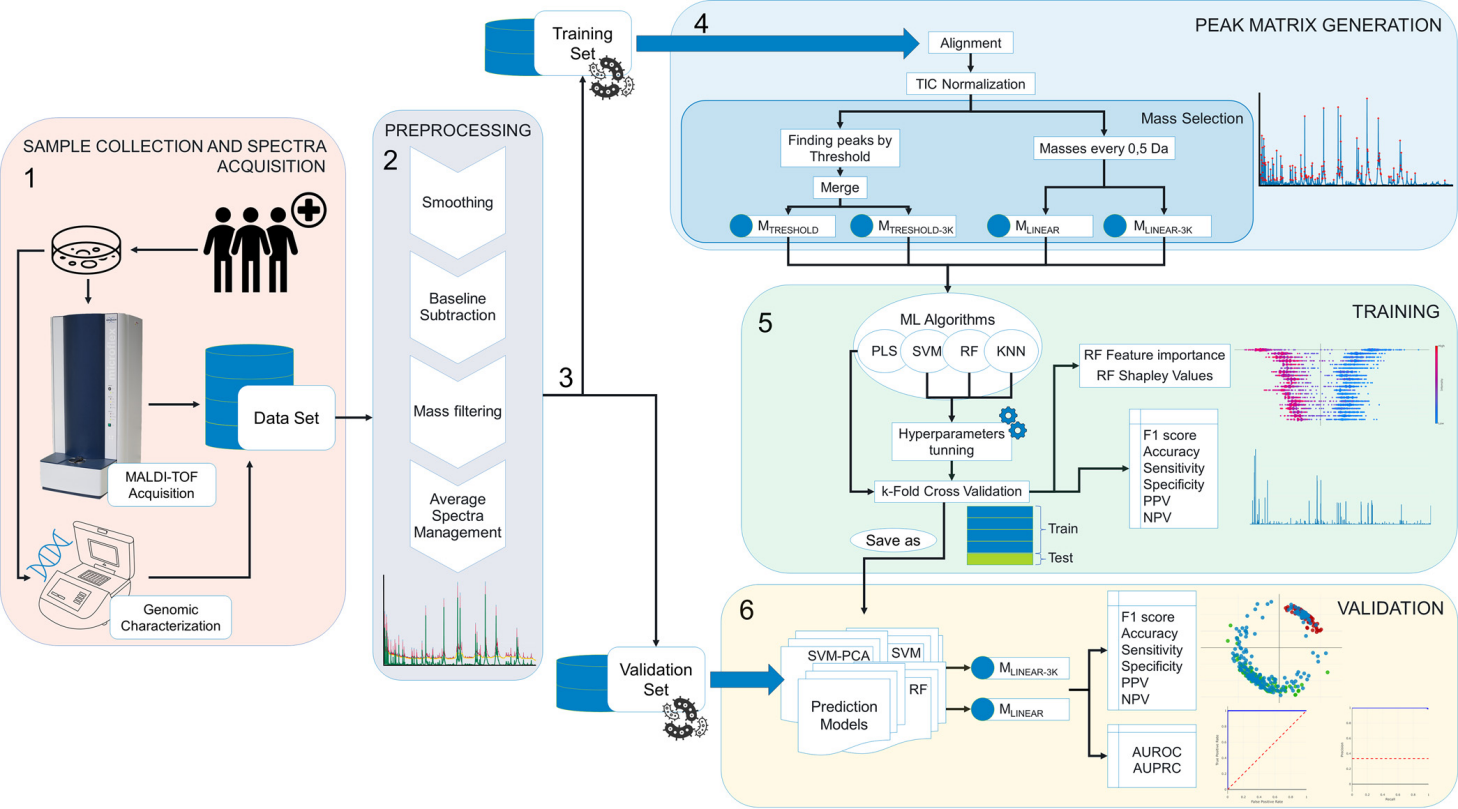
Workflow of MALDI-TOF MS-based CPK prediction. 1. Sample collection and spectra acquisition. Samples are taken from infected patients and pathogens are cultured and further genotypically characterized. The same isolates are analyzed via MALDI-TOF MS. 2. Preprocessing. The mass spectra profiles are extracted from the MALDI-TOF MS and preprocessed using the Clover MS data analysis software package by applying smoothing, baseline subtraction, mass filtering, and average spectra management. 3. Data splitting. The data are split into training and validation sets. The data are labeled as CPK or NCPK as well as by the type of carbapenemase. Information regarding the sequence type, sample type, and location of origin is defined. 4. Peak matrix generation. Spectra are aligned and normalized via the TIC method. Two different mass selection methods are applied (M_THRESHOLD_ and M_LINEAR_) in two different mass ranges: one from 2,000 to 20,000 *m/z* and the other from 3,000 to 20,000 *m/z*. 5. Training. The peak matrices were used as the input data to four supervised machine learning algorithms: partial least squares discriminant analysis (PLSDA), support vector machine (SVM) with and without a principal components analysis (PCA), k-nearest neighbor (KNN) with and without a neighbourhood components analysis (NCA), and random forest (RF). The algorithms were trained and the hyperparameters were optimized. The training steps were then evaluated by calculating the resulting metrics from a k-fold cross-validation method. Once the algorithms were trained and evaluated, a prediction model for each method and peak matrix was built. The metrics reported are the accuracy, the F1 score, the sensitivity, the specificity, and the positive and negative prediction values. The contributions of individual features to the CPK prediction were determined using feature importance and Shapley values. 6. Validation. The evaluation metrics are the same as those used in the training set, but predictive performance is also measured using AUROC and AUPRC. The use of MALDI-TOF MS coupled with machine learning algorithmic processing enables the identification of CPK with an accuracy of 97.83% and of the type of carbapenemase with an accuracy of 95.24%.

The MALDI-TOF MS and data processing workflow is summarized in Fig. 1. Bacterial isolates in the training set were stored at −80°C in a small vial with glass cryopearls (Deltalab, Barcelona, Spain). When required, the isolates were thawed on a blood agar plate (Becton, Dickinson, Madrid) by removing one of the pearls from the tube with a sterile loop and then rolling it on the surface of the agar. The plate was incubated for 18 h, and the isolates were then subcultured for another 18 h on a blood agar plate for an analysis under standard conditions. The plates were incubated in an aerobic atmosphere at 37°C. All of the isolates that were analyzed were of the same age to control for senescence-associated changes in the mass peak spectra. The isolates were subjected to a modified Hodge test to check for the presence of the carbapenemase enzyme. No discordant results were found in relation to the phenotypic and genotypic annotations. The same operator analyzed all of the isolates in the training set to reduce the associated variability.

The bacterial isolates in the validation set were analyzed without standardized conditions of incubation. To improve the existing methodology and with the aim of designing a procedure using universal sample media, the validation stage was first carried out with different culture media. Mass spectra profiles are subject to variations and differences related to the culture medium, and these are caused by biological differences in bacterial growth due to the different nutrients contained in the medium. Although these differences do not influence correct identification by commercial MALDI-TOF manufacturers (21), we wanted to illustrate the challenges and limits of mass spectra-based antimicrobial resistance prediction. Hence, we studied whether CPK classification could be performed by starting off with different bacterial culture media (blood agar, chocolate agar, and MacConkey agar). The culture age was not taken into account. Up to three operators randomly performed the analysis of the validation set.

The bacterial proteome was analyzed via “in-target” protein extraction (31). Briefly, this consists of the direct extraction of protein from the MALDI target by spotting 1 μL of formic acid on the dried sample and then adding the IVD HCCA-portioned matrix (Bruker Daltonics, Germany). Nine replicates (three spots with three spectra each) of each isolate were processed in the training set, and one replicate (one spot with one spectrum) was processed in the validation set.

The MALDI-TOF spectra were acquired in a Microflex LT/SH SMART mass spectrometer with FlexControl software 3.4 in the linear positive ion mode, within a mass range of 2 to 20 kDa. For each spectrum, a total of 240 satisfactory laser shots were acquired in 40 steps by using small spiral motions. External calibration was performed using the bacterial test standard (BTS, Bruker Daltonics, Germany) prior to each run. The MALDI Biotyper Compass software package (v. 4.1.100, Bruker Daltonics) was used to confirm the species by comparison with the mass-spectrum library, and a score above 2.0 was required for the analysis of carbapenemase resistance.

The spectra were preprocessed with Clover MS Data Analysis Software (Clover Biosoft, Granada). The first step involved preprocessing all spectra by applying noise reduction with the Savitzky-Golay filter (smoothing filter: window length, 11; polynomial order, 3) and then subtracting the baseline with the Top-Hat filter (baseline removing filter: factor, 0.02). For model construction, an average spectrum was obtained for each isolate in the training set via a consecutive, two-step process of alignment and merging. This minimizes the variability between replicates of the same isolate. The first step consisted of aligning the replicated spectra of each spot and then obtaining a unique average spectrum per spot. The second step involved repeating this process but aligning the spectra obtained for each replicate spot to produce the final average spectrum. Once this process was completed, only one average spectrum remained for each isolate, and all of them were aligned with each other (shift medium; linear tolerance, 900 ppm; constant tolerance, 3 Da). All of the alignment processes were performed by considering the most representative peaks of each sample included in the set to be aligned. These peaks were then used to create a reference peak list. Each spectrum peak was shifted within a linear tolerance of 2,000 ppm to correspond to this list.

After preprocessing and forming the average spectra for each isolate from the training set, two different mass selection methods were applied in two different ranges: 2,000 to 20,000 *m/z* (M_THRESHOLD_ and M_LINEAR_) and 3,000 to 20,000 *m/z* (M_THRESHOLD-3K_ and M_LINEAR-3K_). Thus, a total of four peak matrices were obtained. The peak matrices from the M_THRESHOLD_ and M_THRESHOLD-3K_ methods were generated by applying a threshold algorithm of value 0.01 so that peaks with at least 1% of the maximum intensity of the spectrum were considered for both ranges. All of the resultant peaks were merged into a common list within a linear tolerance of 600 ppm and a constant tolerance of 3 Da. The matrices from the M_LINEAR_ and M_LINEAR-3K_ methods were obtained by merging the entire spectrum of each isolate in a mass list with values every 0.5 Da for either range. The peak matrices were subsequently normalized by the total ion current (TIC) method. When applying this method, each intensity value was divided by the area under the spectrum value.

### Machine learning algorithms for the identification of CPK isolates.

After selection of the mass spectra, we obtained four different matrices by using two training sets for each method: M_THRESHOLD_, M_THRESHOLD-3K_, M_LINEAR_, and M_LINEAR-3K_. These peak matrices were used as input data for the four supervised machine learning algorithms that were then applied to these matrices: partial least squares discriminant analysis (PLSDA), support vector machine (SVM) with and without a principal components analysis (PCA) applied, k-nearest neighbor (KNN) with and without a neighborhood components analysis (NCA) applied, and random forest (RF). The algorithms were trained, and their hyperparameters were optimized (when applicable) (Table S2). The training steps were then evaluated by calculating the resulting metrics from a k-fold cross-validation method. Once the algorithms were trained and evaluated, prediction models were then built for each method and peak matrix. These prediction models were validated using an external validation set. The samples in the validation set were preprocessed, following the same procedure described for the training set. The validation samples were used as the input data for classification by the prediction models by applying the respective mass selection methods. These samples were previously categorized to compare (and thereby evaluate) the results obtained for the prediction models versus their actual categories.

The study was conducted in two steps. The first step discriminated CPK isolates, and the second step differentiated the different carbapenemase types: *bla*_OXA-48-like_, *bla*_KPC_, *bla*_NDM_, and *bla*_VIM_.

### Evaluation metrics and statistical analysis.

For each of the 24 analytical combinations, we report the main metrics that were used to evaluate the performance. For the internal evaluation in the training, we used a 10-fold cross-validation (k = 10) for CPK differentiation. In the second step, a limited number of samples in the two minority categories were available for the differentiation of the type of carbapenemase, and we therefore implemented a 7-fold cross-validation (k = 7) procedure (32).

The following performance metrics are reported: accuracy, expressed by the fraction of correct classifications; the harmonic mean of precision and sensitivity, expressed as the F1 score; the sensitivity; and the specificity. Features importance was also reported for the RF algorithm of analysis in the discrimination of the CPK isolates. Their contributions were analyzed in two ways: by directly obtaining the values of features importance as one of the outputs of the RF function and by calculating the Shapley values. In the first method, higher values of feature importance indicate greater contributions to splitting the samples in the classifier trees. The importance of a feature is computed (the normalized values of the array sum to 100%) as the total reduction of the criterion brought by that feature. This is also known as the Gini importance (33). Shapley values measure the feature importance in a multivariate way. Thus, the higher the feature position is in the list, the more important it is for discriminating the positive category. The predictor uses either the presence of a high intensity value or the absence of any measured intensity for the positive category.

For validation, we also report the area under the receiver operating characteristic (AUROC) curve as one of the main performance metrics, and both the average precision (AP) and the area under the precision-recall curve (AUPRC) were used to characterize the value under the precision-recall curve. The AUROC indicates the true-positive rate (i.e., the true positive rate against the false-positive rate), and it was used to evaluate the discriminatory ability of the model. The value directly indicates the ability of the model to discriminate between pairs of classes (i.e., higher values indicate greater ability). On the other hand, the AUPRC or AP-PRC indicates the recall against the precision (i.e., the ability to correctly detect the samples from the positive category while minimizing the percentage of false positives). The AUPRC is highly rated in the case of unbalanced data sets, as AUROC does not reflect the performance relative to the precision (or positive predictive value). Therefore, the AUROC value can be high while the precision is low. In the present case (324 CPK/391 NCPK), both data groups were almost balanced; however, the importance of precision in the clinical setting makes this tool valuable for our analysis.

## RESULTS

### Bacterial isolates.

The 715 isolates represent nongeographically related samples from 15 different hospitals throughout Spain. Among the CPK isolates, 37 different sequence types (STs) were detected. Of the 324 CPK isolates, 228 carried the *bla*_OXA-48_ gene, whereas 82 harbored the *bla*_KPC_ gene, 7 carried the *bla*_NDM_ gene, and 7 carried the *bla*_VIM_ gene. These data are summarized in Table S1.

Of the 715 representative isolates, the 479 that were used as the training set were comprised of 246 CPK isolates and 233 NCPK isolates. The CPK isolates included 170 OXA-48-producing isolates, 62 KPC-producing isolates, 7 NDM-producing isolates, and 7 VIM-producing isolates (Table S1). Isolates were randomly selected from the overall database, except for the NDM-producing and VIM-producing isolates, which were purposely selected to form part of the training set because of the small numbers of isolates in both groups. These isolates were examined in triplicate in 3 different spots, thereby yielding the 4,311 spectra that form the database.

The 236 remaining isolates that were used as the validation set were comprised of 78 CPK and 158 NCPK isolates. The CPK isolates included 58 OXA-48-producing isolates and 20 KPC-producing isolates. The 236 spectra that were obtained were further analyzed for the clinical validation of the procedure. Isolates were obtained from different types of samples: biopsy specimen (1), catheter (1), bile (2), peritoneal fluid (2), abscesses (4), respiratory samples (13), wounds (17), blood cultures (19), urine samples (171), and samples of unknown origin (6).

### Machine learning for MALDI-TOF mass spectrometry-based CPK prediction.

**(i) Training.** High kappa values (>80) were obtained for all 24 combinations of methods and analytical algorithms for both steps (32): the detection of CPK and the subsequent differentiation of the carbapenemase type (Table S3). The evaluation of the M_LINEAR_ and the M_THRESHOLD_ methods showed that in the CPK detection step, the accuracy of the M_LINEAR_ method was, on average, higher, with the correct identification of 97.95% of CPK isolates (compared with 96.44% being correctly identified by the M_THRESHOLD_ method). The evaluation of the predictive performance of the algorithms (PLS, SVM, PCA-SVM, KNN, NCA-KNN, and RF), averaging all of the methods (M_LINEAR_ and M_THRESHOLD_), showed that the RF algorithm performed best, (accuracy of 99.27%). The evaluation of the influence of the mass range in the analysis revealed that taking into account all of the mass peaks in the spectra from 2 to 20 kDa, produced excellent metric performance (i.e., the M_LINEAR_ method combined with the RF algorithm provides the best metrics [accuracy of 99.79%, sensitivity of 100%, and specificity of 99.57%]).

In the second step, the best metrics were also found in the M_LINEAR_ method, yielding a correct identification of 87.85% isolates (versus 86.65% with the M_THRESHOLD_ method). The evaluation of the predictive performance of the five analytical algorithms (PLS, SVM, KNN, NCA-KNN, and RF), averaging all of the methods (M_LINEAR_ and M_THRESHOLD_), showed that the RF algorithm performed best (accuracy of 90.39%). In this step, the SVM and PCA combination was omitted, as clustering was not possible in the prior PCA analysis. According to our results, the classifier that therefore proved capable of differentiating between carbapenemases in K. pneumoniae was the RF algorithm with the M_LINEAR_ method, which yielded excellent performance metrics, with 90.91% accuracy for either the complete mass range of analysis or using the mass peaks from 3 kDa onwards, only.

**FIG 2:**
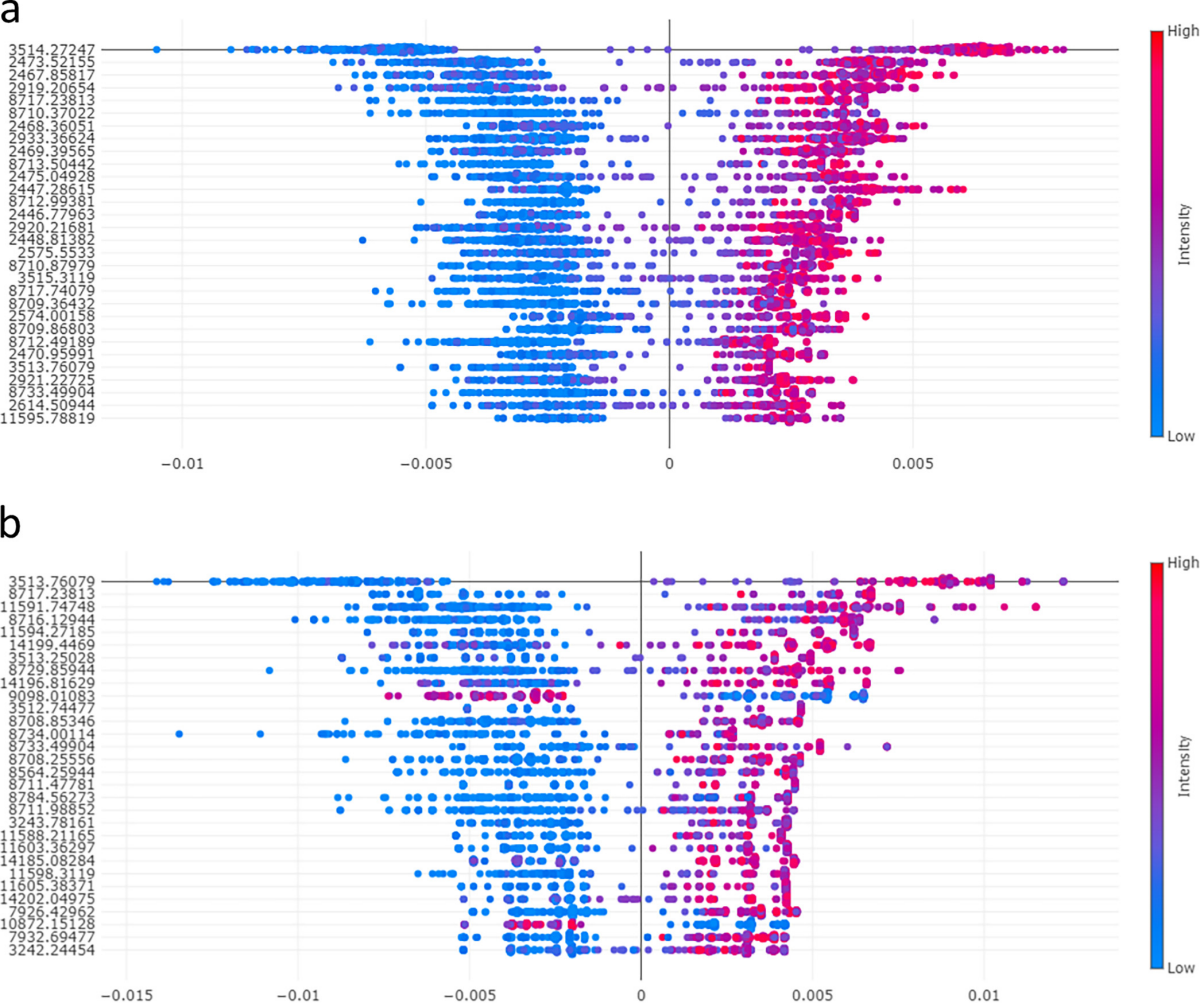
Quantification of feature impact on the RF prediction for CPK through an analysis of the Shapley values of the 30 most important features. The images represent the quantification of feature impact on the RF prediction for the (a) M_LINEAR_ and (b) M_LINEAR-3k_ methods for the prediction of CPK through the analysis of the Shapley values. The values on the left represent the most impactful features (*m/z* peak location) on the RF prediction that is the mean Shapley value. The scatterplot on the right indicates the distribution of the Shapley values and their impacts on the model output across all test isolates in the validation set. The colors of each test spectrum are represented as a dot (blue for a low feature value and red for a high feature value) to indicate the feature value (i.e., the intensity of the respective feature in the spectrum). Shifts to the right in the test spectra represented as dots indicate greater contributions of the features (*m/z* peak) to the category selected as positive (CPK).

Then, we determined the feature importance and the Shapley values for evaluating the CPK prediction performance of the RF algorithm. The average and per data point Shapley values for the 30 features with the highest average contributions are shown in Fig. 2. For each feature (each row) there is one point (Shapley value) per sample. Shifts of the points to the right indicate greater contributions to the category selected as positive (CPK). In Fig. 2, the colors of the tails of the distribution plots for each feature indicate either the highest (red) or lowest (blue) feature value. Therefore, we can see that the RF algorithm uses either the presence of a high intensity value (red) or the absence of any measured or low intensity (blue) for CPK prediction. Most of the masses, (especially in the M_LINEAR_ method) that are important for the Shapley analysis are those that occur at high intensities for CPK prediction. The mass peak at 3,514 Da made a large contribution for the prediction of CPK, and it is represented in both methods. Not having analyzed to a large extent the protein sequence by MS/MS, the theoretical identification of the protein is not related to carbapenem resistance but to a glycosidase (Uniprot: A0A2V1LFY6), making the pattern matching even more important (17). This mass peak is distributed independently of the ST of the isolate, as it is a high intensity mass peak area in all of the CPK that were analyzed.

**FIG 3:**
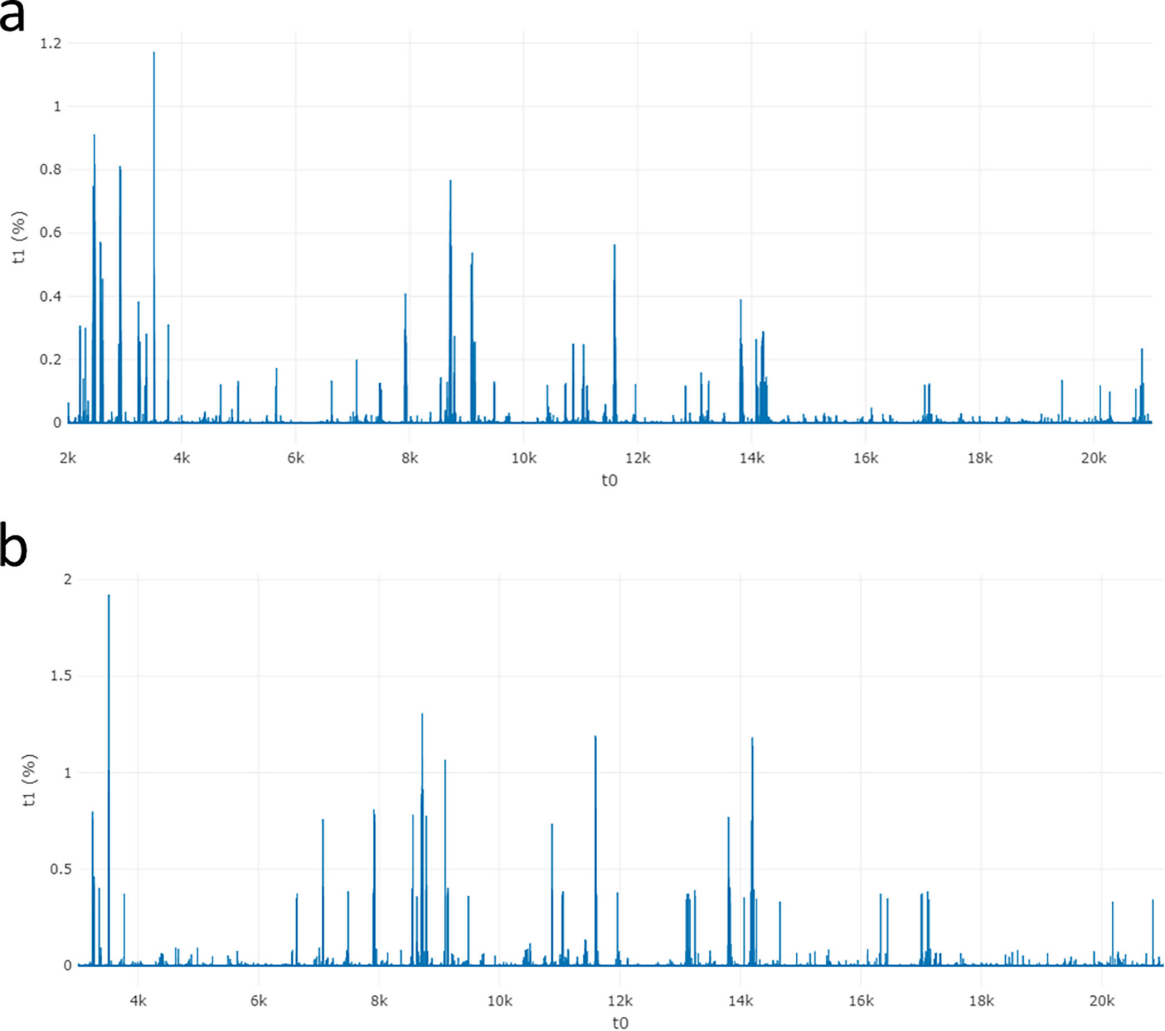
Bar plot of feature importance for the RF algorithm in the CPK prediction. The graph represents the importance of each mass peak for the result of the RF analysis in the cases of the (a) M_LINEAR_ and (b) M_LINEAR-3K_ methods. The *x* axis represents the individual sample masses. The *y* axis represents the percentage of importance of each mass peak for the decision making in the allocation of each isolate into one of the analytical categories (CPK or NCPK).

Besides, the analysis revealed that the presence or absence of any feature is by itself not relevant in the classification of CPK, and the importance of any particular feature is balanced between being or not being a CPK for either method. This finding justifies the importance of analyzing a series of mass peaks and not only specific mass peaks. Furthermore, in the M_LINEAR_ method, we observed that almost all (29/30) of the feature bins with the highest average impact are feature bins with a mass-to-charge ratio (*m/z*) value of less than 10,000 Da. In the case of the M_LINEAR-3K_, the proportion decreased to 19 of the 30 feature bins with the highest impacts in the prediction. This can be attributed to the fact that 15 of the 30 feature bins that were used in the M_LINEAR_ method have masses below 3,000 Da, meaning that the M_LINEAR-3K_ method can explore other mass ranges for classification, being that results are close between both mass ranges. This finding again justifies the importance of analyzing a series of mass peaks and not only specific mass peaks that can act as biomarkers, thereby highlighting the importance of machine learning tools for their capacity of analyzing big data and not focusing on the pure correlation between the presence of a mass peak and a resistance phenotype. The extended bar plot of feature importance (Fig. 3) indicates that in both methods, the classifier uses certain areas across the entire mass range and is capable of adapting to different situations. In this case, the feature importance from 2k to 3k Da in M_LINEAR_ was offset with the other features from 3k Da in the M_LINEAR-3k_ method.

**FIG 4:**
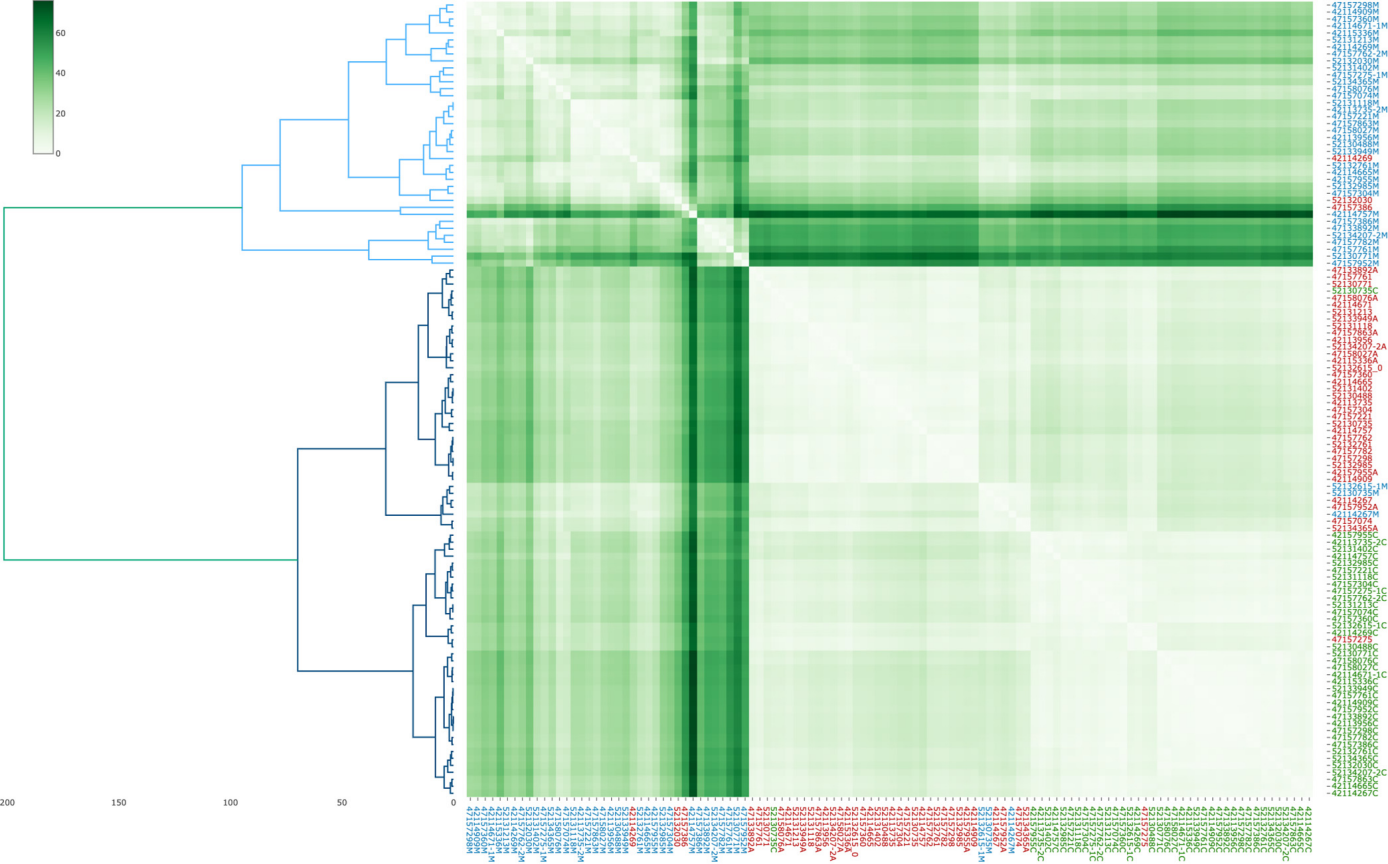
Growth media impact in the bacterial proteome of K. pneumoniae. Hierarchical clustering after applying PCA with a heat map in 114 NCPK in a 0.1 threshold peak matrix. The data were scaled, and the Euclidean distance between samples expressed using a green-colored scale and Ward’s metric. Isolates for which the mass spectra were obtained prior to culture in MacConkey agar are indicated in blue. Isolates cultured on blood agar are indicated in red. Isolates cultured on chocolate agar are indicated in green. The green scale shows the distances between pairs of isolates.

**(ii) Validation.** We first sought to determine whether the use of different sample media affects the prediction of antimicrobial resistance. For this purpose, we tested the data set classifier with clinical samples cultured on blood agar, chocolate agar, and MacConkey agar. We observed poor classification of the isolates collected in both chocolate agar and MacConkey agar (data not shown), highlighting the importance of the culture medium in the predictive performance of the algorithms. We explain this reduction in performance by the difference in the expression of certain peptides and proteins that were determined by the effects of the nutrients on bacterial growth. The hierarchical clustering (HC) of all isolates (*n* = 114) where PCA was first applied revealed three well-defined clusters, with each corresponding to a particular culture medium, as shown in Fig. 4. The image also includes a heat map to illustrate the differences. All isolates are NCPK, and the differences in the classification are fully dependent on the culture medium.

The kappa values were slightly lower for the validation stage than for the training stage, especially when the M_THRESHOLD_ method was applied. In the CPK detection step, the mean accuracy for the correct identification of CPK was 93.60% with the M_LINEAR_ method and 84.71% with the M_THRESHOLD_ method (Table S3). Thus, the level of agreement was high for the M_LINEAR_ method (32), independently of the method of analysis, with a decrease in accuracy of only 4.35%, relative to the data set, representing excellent model performance. In the case of the M_THRESHOLD_ method, the decrease in the accuracy was 11.73% (i.e., more than twice the decrease observed with the M_LINEAR_ method).

The evaluation of the predictive performance of the six analytical algorithms (PLS, SVM, PCA-SVM, KNN, NCA-KNN, and RF) for both methods (M_LINEAR_ and M_THRESHOLD_) and mass ranges showed that the classifier that is most capable of providing precise CPK prediction is the RF algorithm with the M_LINEAR_ method. That is, considering all of the mass peaks in the spectra from 2 to 20 kDa yielded excellent performance metrics, with an accuracy of 97.83%, a sensitivity of 100%, a specificity of 96.73%, and an F1 score of 96.85%.

In this second stage of the differentiation of the carbapenemase type, the algorithms performed even better than in the training data set, with an almost perfect classification in 8 of the 20 method-algorithm combinations. Thus, according to our results, the classifier that was most capable of differentiating between KPC- and OXA-48-producing K. pneumoniae was the RF algorithm when the M_LINEAR_ method was applied, which yielded excellent performance metrics, with an accuracy of 98.70% when the complete mass range was analyzed.

We also report the AUROC and the AUPRC as performance metrics. AUROC can be understood as the probability of correctly classifying a pair of samples (i.e., of the sample being CPK or NCPK), whereas AUPRC quantifies the ability to correctly detect samples from an imbalanced binary classification (CPK/NCPK) while minimizing false results. We decided to exclude the M_THRESHOLD_ method and the KNN and NCA-KNN algorithms from the analysis in further studies, as these performed less well than the other method-algorithm combinations and would not provide any relevant information. We also decided to exclude the analysis of the mass range that takes into account only the mass peaks from 3 kDa onwards, as they provide similar and slightly worse results than does the use of the complete spectra in previous metrics. We analyzed the extent to which the best model (RF) was capable of predicting whether an isolate was a CPK (Fig. S1), observing high overall performance in both AUROC and AUPRC. Detailed information for all method-algorithm combinations is included in Table S4. PLS and RF were the only algorithms that provided outstanding discrimination of CPK isolates, yielding values higher than 0.90 in the AUROC and AUPRC. More specifically, the RF algorithm yielded perfect classification of the CPK isolates, with a value of 1.00 for both AUROC and AUPRC (32). For the second stage of the differentiation of the carbapenemase type, as only NDM-producing and VIM-producing isolates were used to form part of the training set, owing to the small numbers of isolates in both groups, the algorithm was only validated for the prediction of OXA-48-like- and KPC-producing isolates. In this second stage, the SVM and the RF algorithm provided outstanding discrimination of the CPK isolates. The RF algorithm again performed best, with an AUPRC value of 0.99 for OXA-48-like producing isolates and an AUROC value of 0.978 with the M_LINEAR_ method. For KPC-producing isolates, the AUPRC was 0.97 and the AUROC was 0.975.

## DISCUSSION

The analysis of the operating procedure and subsequent data management are critical for the reliable recovery of information from mass spectrum profiles that are obtained by MALDI-TOF MS. In a previous study, we analyzed the biological and technical reproducibility of the operating procedure and evaluated how this variance affected the final results, and we presented an improved MALDI-TOF MS data analysis pipeline for the identification of CPK (25). In the present study, we used a larger collection of K. pneumoniae isolates to validate the method so that it can be fully integrated into the normal workflow in clinical microbiological laboratories. We have demonstrated that the MALDI-TOF mass spectra-based prediction of CPKs from routine diagnostic clinical samples can provide accurate predictions within 24 h of sample collection.

Weis et al. reported that the performance of classifiers trained on mass spectra from one site is not generalizable to mass spectra measured at other sites, as the analysis can be influenced by many sources, including different phylogenetic strains, different prevalence of resistance, technical variability, and different machine-specific parameters and settings (20). Both the aforementioned study and the present study are restricted to a particular country (Switzerland and Spain, respectively). We did not compare the results of the classifier by constructing different databases based on different locations, as the number of spectra included in our study is much lower; however, the accuracy of prediction that was obtained with our database, which was constructed with isolates with different phylogenetic origins (37 STs detected in the study), was 97.83 (i.e., excellent classification). Note that the isolates that were not correctly classified (*n* = 5) were all NCPK isolates that were identified as CPK from the same location (Complejo Hospitalario Universitario de A Coruña, Spain). Thus, the elaboration of the database is an important factor. Exploring the possibility of constructing specific classifiers for smaller areas is a possible solution. Training with the most recent and similar isolates could provide better predictive performance, as variance in the data can, of course, have the potential to decrease the performance of the algorithm. However, in general, large data sets improve the performance of trained algorithms. We cannot guarantee that our constructed MALDI-TOF model fits all of the biological and epidemiological variations of bacteria, as we have not performed a prospective validation over time in different centers. Therefore, we propose the individual, preliminary validation of the method with known, characterized isolates so that, when required, the database can be adapted to individual, regionally specific characteristics with the regular reevaluation of the data set with new isolates. Thus, the method will be trained to recognize changes and adaptations of the evolving bacteria.

The type of medium and other culture conditions have some effects but do not affect the overall ability of MALDI-TOF MS to identify bacterial species, as previously demonstrated (21). However, we observed that the culture media affects the predictive performance of CPK. We must consider that the evaluated isolates should have been grown on the same culture medium (in this case, blood agar) as were the isolates forming the database. This is, of course, a current limitation of the method. Thus, further improvements in the algorithm should be made to minimize the variance produced by the culture medium to improve the clinical applicability of the method.

Few studies have considered full mass spectra, rather than single peaks, for antimicrobial prediction. However, the negative impact of the peak prediction of resistance phenotypes has already been reported (34, 35). The reliance on single biomarkers that may be related to a resistance phenotype but cannot be directly linked to a functional characteristic of the isolate is a drawback of the method. In addition, the variability associated with a single biomarker is higher, especially as proteins lack constant specific expression (36). Many proteins causing resistance are beyond the effective mass range of MALDI-TOF mass spectra. For example, β-lactamases in K. pneumoniae weigh approximately 30 kDa. Therefore, we propose an algorithm based on pattern matching with resistance-associated changes produced in the bacteria by using the complete proteome and not only specific mass peaks. We also assessed whether predictive performance is primarily driven by only a subset of peaks or whether the full spectrum is used. The Shapley values indicate that several mass peaks (rather than a particular mass peak) contribute to the identification of CPK. The proteome analysis is started at 3 kDa to minimize the impact of the noise in the 2 to 3 kDa mass range, which is usually greater in that part of the spectrum. However, the similarity between the results obtained using the M_LINEAR_ and the M_LINEAR-3K_ methods reveals that the potential impact of the noise is removed by the algorithm and highlights its adaptability to different spectra. Moreover, the Shapley values indicate that extremely high or low feature values (corresponding to the presence or absence of a MALDI-TOF mass peak) contribute to the prediction outcome, rather than smaller variations in the feature magnitude. This is consistent with our results for the M_LINEAR_ and the M_LINEAR-3K_ methods, confirming that the detection of proteins is responsible for the predictive power, rather than confounding signals with noise (20). Therefore, the study results support the proposed analysis of a series of mass peaks, rather than only specific peptides or proteins that can act as biomarkers. Focusing on pattern matching, rather than on core peaks, would improve the reliability of the identification of resistance in bacteria by using well-constructed databases, rather than species-specific peak constant expression, which have been shown to be nonexistent.

The prediction of the carbapenemase type yielded an accuracy of 95.24%. The inclusion of other VIM and NDM-type carbapenemases would enable the validation of the method for these groups of carbapenemases in the clinical setting. Regarding OXA-48 and KPC-type carbapenemases, the model exhibits great performance with AUROC and AUPRC values of higher than 0.95 for both types of enzymes, regardless of the date set being unbalanced, favoring OXA-48-type identification performance due to the higher number of isolates of this group (228 OXA-48 versus 82 KPC). Globally, ST11, ST14, ST101, ST147, and ST258/512 are the major carbapenemase-producing K. pneumoniae clones (37). In our study, 37 STs were detected. The results of the clonal structure of the CPK isolates revealed that ST-15, ST-147, and ST-392 are exclusively expressed in the carbapenemase OXA-48, whereas ST-512 is linked to KPC in all isolates. Other clones, such as ST-307, are similarly distributed in both carbapenemase types, and ST-11, although represented in both groups, is predominant in OXA-48 isolates. The great variability of the clones among both groups and the fact that some clones carry both carbapenemase types justifies the idea of the excellent performance classification of MALDI-TOF MS. The determination of the type of carbapenemase in a specific epidemiological setting, such as in areas with a high prevalence of OXA-48 or KPC-producing K. pneumoniae isolates, would be helpful until molecular results are available. Thus, MALDI-TOF could contribute to the rapid prescription of targeted treatments, such as the new β-lactam-β-lactamase inhibitors ceftazidime-avibactam and imipenem/relebactam, which are specifically designed to act against carbapenemase-type isolates (38).

In summary, the study findings demonstrate that MALDI-TOF mass spectrometry-based machine learning can provide novel ways by which to predict antimicrobial resistance in highly relevant clinical scenarios, such as the identification of CPK. In our opinion, the ideal application for this MALDI-TOF-based technique would be as an initial screening test for the identification of CPK isolates in clinical laboratories, as direct tracking can be performed at the same time as bacterial identification, using the same hands-on procedure and the same spectra. The only difference would be the posterior processing by machine learning algorithms, which takes only minutes to apply, as the only extra step needed is the uploading of the spectra in an analysis platform that is configured with the above-mentioned algorithms. The bioinformatics processing is fully automated. Therefore, no particular skills beyond the basic use of MALDI-TOF MS are needed. This enables the full integration of the technique in routine laboratory work. A potential future application of the proposed method is the identification of CPK directly from the positive blood culture in cases of bacteremia and/or sepsis due to the high clinical impact of these diseases. Although the prediction of resistance alone would not be used, the shortened time to report a CPK in blood cultures may support clinical decision-making, together with antimicrobial stewardship programmes, thereby significantly reducing the time to therapeutic de-escalation (39). This approach also has huge potential for expansion to other combinations of bacterial identification/resistance detection. In a potential outbreak situation, the MALDI-TOF MS-based identification of CPK isolates might provide the first evidence indicating the need to initiate intensive health care and infection control measures, thereby altering the clinical outcomes of these infections.
