# Supplementary material for: Direct Detection of Carbapenemase-Producing Klebsiella pneumoniae by MALDI-TOF Analysis of Full Spectra Applying Machine Learning
Source: J Clin Microbiol. 2023 May 18;61(6):e01751-22. doi: 10.1128/jcm.01751-22 (PMC10281162; doi:10.1128/jcm.01751-22)
Supplement: Supplemental file 1 — Supplemental material. Download jcm.01751-22-s0001.pdf, PDF file, 0.78 MB [file jcm.01751-22-s0001.pdf]

Supplementary material.

Table 1. Detailed information for all isolates used in the study.

a. Training

Isolate	Sequencing code	ST	Resistance mechanism	Location
1	AI2583	11	OXA-48	Hospital Universitario Gregorio Marañón (Madrid)
2	AI2585	405	OXA-48	Hospital Universitario Gregorio Marañón (Madrid)
3	AI2586	11	OXA-48	Hospital Universitario Gregorio Marañón (Madrid)
4	AI2587	11	OXA-48	Hospital Universitario Gregorio Marañón (Madrid)
5	AI2588	512	KPC-3	Hospital Universitario Gregorio Marañón (Madrid)
6	AI2589	11	OXA-48	Hospital Universitario Gregorio Marañón (Madrid)
7	AI2590	11	OXA-48	Hospital Universitario Gregorio Marañón (Madrid)
8	AI2818	15	OXA-48	Hospital Universitario Gregorio Marañón (Madrid)
9	AI2819	15	OXA-48	Hospital Universitario Gregorio Marañón (Madrid)
10	AI2593	219	OXA-48	Hospital Universitario Gregorio Marañón (Madrid)
11	AI2594	15	OXA-48	Hospital Universitario Gregorio Marañón (Madrid)
12	AI2595	405	OXA-48	Hospital Universitario Gregorio Marañón (Madrid)
13	AI2596	11	OXA-48	Hospital Universitario Gregorio Marañón (Madrid)
14	AI2861	11	OXA-48	Hospital Universitario Gregorio Marañón (Madrid)
15	AI2597	11	OXA-48	Hospital Universitario Gregorio Marañón (Madrid)
16	AI2600	405	OXA-48	Hospital Universitario Gregorio Marañón (Madrid)
17	AI2822	392	OXA-48	Hospital Universitario Gregorio Marañón (Madrid)
18	AI2823	11	OXA-48	Hospital Universitario Gregorio Marañón (Madrid)
19	AI2601	11	OXA-48	Hospital Universitario Gregorio Marañón (Madrid)
20	AI2824	11	OXA-48	Hospital Universitario Gregorio Marañón (Madrid)
21	AI2825	11	OXA-48	Hospital Universitario Gregorio Marañón (Madrid)
22	AI2603	11	OXA-48	Hospital Universitario Gregorio Marañón (Madrid)
23	AI2604	11	OXA-48	Hospital Universitario Gregorio Marañón (Madrid)
24	AI2605	11	OXA-48	Hospital Universitario Gregorio Marañón (Madrid)
25	AI2607	392	OXA-48	Hospital Universitario Gregorio Marañón (Madrid)
26	AI2829	15	OXA-48	Hospital Universitario Lucus Augusti (Lugo)
27	AI2830	147	NDM-1	Hospital Universitario Lucus Augusti (Lugo)
28	AI2621	405	OXA-48	Hospital Universitari Germans Trias i Pujol (Badalona)
29	AI2622	405	OXA-48	Hospital Universitari Germans Trias i Pujol (Badalona)
30	AI3021	15	OXA-48	Hospital General de Alicante (Alicante)
31	AI2626	11	VIM-1	Hospital Puerta del Mar (Cádiz)

32	AI2835	512	KPC-3	Hospital Puerta del Mar (Cádiz)
33	AI2627	512	KPC-3	Hospital Puerta del Mar (Cádiz)
34	AI2628	512	KPC-3	Hospital Puerta del Mar (Cádiz)
35	AI2630	15	OXA-48	Hospital Puerta del Mar (Cádiz)
36	AI2631	512	KPC-3	Hospital Puerta del Mar (Cádiz)
37	AI2836	512	KPC-3	Hospital Puerta del Mar (Cádiz)
38	AI2632	512	KPC-3	Hospital Puerta del Mar (Cádiz)
39	AI2635	392	OXA-48	Hospital Clinic (Barcelona)
40	AI2838	307	OXA-48	Hospital Clinic (Barcelona)
41	AI2636	392	OXA-48	Hospital Clinic (Barcelona)
42	AI2639	392	OXA-48	Hospital Clinic (Barcelona)
43	AI2640	392	OXA-48	Hospital Clinic (Barcelona)
44	AI2641	392	OXA-48	Hospital Clinic (Barcelona)
45	AI2842	1401	OXA-48	Hospital Clinic (Barcelona)
46	AH0326	392	OXA-48	Hospital Clinic (Barcelona)
47	AI2844	198	OXA-48	Hospital Clinic (Barcelona)
48	AI2846	45	OXA-48	Hospital Clinic (Barcelona)
49	AI2847	307	OXA-48	Hospital Clinic (Barcelona)
50	AI2848	1083	VIM-1	Hospital Clinic (Barcelona)
51	AH0327	392	OXA-48	Hospital Clinic (Barcelona)
52	AI2850	151	OXA-48	Hospital Universitario Mutua de Terrassa (Terrassa)
53	AI2851	152	OXA-48	Hospital Universitario Mutua de Terrassa (Terrassa)
54	AI2856	147	OXA-48	Hospital Universitario Mutua de Terrassa (Terrassa)
55	AI2862	405	OXA-48	Hospital Universitario La Paz (Madrid)
56	AI2867	39	VIM-1	Hospital Universitario La Paz (Madrid)
57	AI2868	307	OXA-48	Hospital Universitario La Paz (Madrid)
58	AI3024	15	OXA-48	Hospital Universitario La Paz (Madrid)
59	AI2870	11	OXA-48	Hospital Universitario Ramón y Cajal (Madrid)
60	AI2643	307	KPC	Hospital Universitario Ramón y Cajal (Madrid)
61	AI2873	11	OXA-48	Hospital Universitario Ramón y Cajal (Madrid)
62	AI2874	11	OXA-48	Hospital Universitario Ramón y Cajal (Madrid)
63	AI2875	11	OXA-48	Hospital Universitario Ramón y Cajal (Madrid)
64	AI2876	11	OXA-48	Hospital Universitario Ramón y Cajal (Madrid)
65	AI2644	307	KPC-3	Hospital Universitario Ramón y Cajal (Madrid)
66	AI2878	11	OXA-48	Hospital Universitario Ramón y Cajal (Madrid)

67	AI2879	11	OXA-48	Hospital Universitario Ramón y Cajal (Madrid)
68	AI2880	101	NDM-1	Hospital Universitario Ramón y Cajal (Madrid)
69	AI3025	15	VIM-1	Hospital Universitario Ramón y Cajal (Madrid)
70	AI2882	307	OXA-48	Hospital Universitario Ramón y Cajal (Madrid)
71	AI2645	307	KPC-3	Hospital Universitario Ramón y Cajal (Madrid)
72	AI2885	307	VIM-1	Hospital Universitario Ramón y Cajal (Madrid)
73	AI2647	307	KPC-3	Hospital Universitario Ramón y Cajal (Madrid)
74	AI2892	11	OXA-48	Hospital Universitario Ramón y Cajal (Madrid)
75	AI2648	307	KPC-3	Hospital Universitario Ramón y Cajal (Madrid)
76	AI2893	45	OXA-48	Hospital Universitario Ramón y Cajal (Madrid)
77	AI2895	11	OXA-48	Hospital Universitario Ramón y Cajal (Madrid)
78	AI2649	307	KPC	Hospital Universitario Ramón y Cajal (Madrid)
79	AI2896	307	OXA-48	Hospital Universitario Ramón y Cajal (Madrid)
80	AI2897	11	OXA-48	Hospital Universitario Ramón y Cajal (Madrid)
81	AI2650	307	KPC-3	Hospital Universitario Ramón y Cajal (Madrid)
82	AI2651	459	KPC-3	Hospital Universitario Ramón y Cajal (Madrid)
83	AI2898	101	NDM-1	Hospital Universitario Ramón y Cajal (Madrid)
84	AI2900	11	OXA-48	Hospital Universitario Ramón y Cajal (Madrid)
85	AI2652	307	KPC-3	Hospital Universitario Ramón y Cajal (Madrid)
86	AI2653	307	KPC-3	Hospital Universitario Ramón y Cajal (Madrid)
87	AI2901	307	KPC-3	Hospital Universitario Ramón y Cajal (Madrid)
88	AI2654	307	KPC	Hospital Universitario Ramón y Cajal (Madrid)
89	AI2902	11	OXA-48	Hospital Universitario Ramón y Cajal (Madrid)
90	AI2655	307	KPC-3	Hospital Universitario Ramón y Cajal (Madrid)
91	AI3027	3362	OXA-48	Complejo Hospitalario de Jaén (Jaén)
92	AI2906	392	OXA-48	Hospital Universitario La Princesa (Madrid)
93	AI2907	5001	OXA-48	Hospital Universitario La Princesa (Madrid)
94	AI2908	39	OXA-48	Hospital Universitario La Princesa (Madrid)
95	AI2909	11	VIM-1	Hospital Universitario La Princesa (Madrid)
96	AI2911	392	OXA-48	Hospital Universitario La Princesa (Madrid)
97	AI2912	392	OXA-48	Hospital Universitario La Princesa (Madrid)
98	AI2914	11	OXA-48	Hospital Universitario La Princesa (Madrid)
99	AI3028	392	OXA-48	Hospital Universitario La Princesa (Madrid)
100	AI2917	392	OXA-48	Hospital Universitario La Princesa (Madrid)
101	AI2919	11	OXA-48	Hospital Universitario La Princesa (Madrid)

102	AI2922	11	OXA-48	Hospital Universitario La Princesa (Madrid)
103	AI2923	392	OXA-48	Hospital Universitario La Princesa (Madrid)
104	AI2925	340	OXA-48	Hospital Universitario La Princesa (Madrid)
105	AI2926	11	OXA-48	Hospital Universitario La Princesa (Madrid)
106	AI2927	392	OXA-48	Hospital Universitario La Princesa (Madrid)
107	AI2929	392	OXA-48	Hospital Universitario La Princesa (Madrid)
108	AI2931	392	OXA-48	Hospital Universitario La Princesa (Madrid)
109	AI2932	392	OXA-48	Hospital Universitario La Princesa (Madrid)
110	AI3030	307	OXA-48	Hospital Universitario La Princesa (Madrid)
111	AI2934	395	NDM-1	Hospital Virgen de la Macarena (Sevilla)
112	AI2940	258	KPC-3	Hospital Universitario Son de Espases (Palma de Mallorca)
113	AI2660	11	VIM-1	Hospital Universitario Son de Espases (Palma de Mallorca)
114	AI2669	15	OXA-48	Hospital Universitario Central de Asturias (Oviedo)
115	AI2670	405	OXA-48	Hospital Universitario Central de Asturias (Oviedo)
116	AI2671	147	OXA-48	Hospital Universitario Central de Asturias (Oviedo)
117	AI2675	326	OXA-48	Hospital Universitario Central de Asturias (Oviedo)
118	AI2676	147	OXA-48	Hospital Universitario Central de Asturias (Oviedo)
119	AI2677	405	OXA-48	Hospital Universitario Central de Asturias (Oviedo)
120	AI2678	147	OXA-48	Hospital Universitario Central de Asturias (Oviedo)
121	AI2681	326	OXA-48	Hospital Universitario Central de Asturias (Oviedo)
122	AI2682	147	OXA-48	Hospital Universitario Central de Asturias (Oviedo)
123	AN2337	147	OXA-48	Hospital Universitario Central de Asturias (Oviedo)
124	AI3034	4387	OXA-48	Hospital Universitario Central de Asturias (Oviedo)
125	AI3035	147	OXA-48	Hospital Universitario Central de Asturias (Oviedo)
126	AI2686	326	OXA-48	Hospital Universitario Central de Asturias (Oviedo)
127	AI2687	147	OXA-48	Hospital Universitario Central de Asturias (Oviedo)
128	AH0330	15	OXA-48	Hospital Universitario Central de Asturias (Oviedo)
129	AI2691	147	OXA-48	Hospital Universitario Central de Asturias (Oviedo)
130	AI3037	147	OXA-48	Hospital Universitario Central de Asturias (Oviedo)
131	AI2953	307	OXA-48	Hospital Universitario Central de Asturias (Oviedo)
132	AI2698	15	OXA-48	Hospital Universitario Central de Asturias (Oviedo)
133	AI3039	307	OXA-48	Hospital de Guadalajara (Guadalajara)
134	AI2958	11	OXA-48	Hospital de Guadalajara (Guadalajara)
135	AI2960	307	OXA-48	Hospital de Guadalajara (Guadalajara)
136	AI2962	307	OXA-48	Hospital de Guadalajara (Guadalajara)

137	AI2965	307	OXA-48	Hospital de Guadalajara (Guadalajara)
138	AI2966	307	OXA-48	Hospital de Guadalajara (Guadalajara)
139	AI3040	307	OXA-48	Hospital de Guadalajara (Guadalajara)
140	AI2967	405	OXA-48	Hospital de Guadalajara (Guadalajara)
141	AI2968	307	OXA-48	Hospital de Guadalajara (Guadalajara)
142	AI2969	307	OXA-48	Hospital de Guadalajara (Guadalajara)
143	AI2973	11	OXA-48	Hospital de Guadalajara (Guadalajara)
144	AI2974	5002	OXA-48	Hospital de Guadalajara (Guadalajara)
145	AI2975	307	OXA-48	Hospital de Guadalajara (Guadalajara)
146	AI2976	307	OXA-48	Hospital de Guadalajara (Guadalajara)
147	AI2977	11	OXA-48	Hospital de Guadalajara (Guadalajara)
148	AI3043	11	KPC-2	Hospital de Móstoles (Madrid)
149	AI3044	101	KPC-2	Hospital Fundación de Alcorcón (Madrid)
150	AI3048	11	KPC-2	Hospital de Guadalajara (Guadalajara)
151	AI3049	101	KPC-2	Hospital Fundación de Alcorcón (Madrid)
152	AI3053	11	KPC-2	U*
153	AI3054	11	KPC-2	U*
154	AI3056	1961	KPC-2	U*
155	AN2338	512	KPC-3	U*
156	AN2339	258	KPC-3	U*
157	AI2700	1961	KPC-2	Complejo Hospitalario de Pontevedra (Pontevedra)
158	AI2701	273	KPC-2	Hospital Universitario Gregorio Marañón (Madrid)
159	AI2979	1961	KPC-2	Complejo Hospitalario de Pontevedra (Pontevedra)
160	AI2706	512	KPC-3	Hospital General La Mancha Centro (Ciudad Real)
161	AI2707	512	KPC-3	Hospital General La Mancha Centro (Ciudad Real)
162	AI2709	678	KPC-3	Hospital de El Escorial (Madrid)
163	AI2980	1961	KPC-2	Complejo Hospitalario de Pontevedra (Pontevedra)
164	AI2982	1961	KPC-2	Complejo Hospitalario de Pontevedra (Pontevedra)
165	AI2713	273	KPC-2	Hospital Universitario Gregorio Marañón (Madrid)
166	AI2717	1961	KPC-2	Complejo Hospitalario de Pontevedra (Pontevedra)
167	AI2985	1961	KPC-2	Complejo Hospitalario de Pontevedra (Pontevedra)
168	AI3060	1961	KPC-2	Complejo Hospitalario de Pontevedra (Pontevedra)
169	AI2722	307	KPC-3	Hospital San Joan de Deu (Fiundación Althaia, Manresa)
170	AI2739	11	OXA-48	Hospital Universitario Marqués de Valdecilla (Santander)
171	AI2740	5000	OXA-48	Hospital Universitario Marqués de Valdecilla (Santander)

172	AI2741	5000	OXA-48	Hospital Universitario Marqués de Valdecilla (Santander)
173	AI2743	11	OXA-48	Hospital Universitario Marqués de Valdecilla (Santander)
174	AI2989	11	OXA-48	Hospital Universitario Marqués de Valdecilla (Santander)
175	AI2745	11	OXA-48	Hospital Universitario Marqués de Valdecilla (Santander)
176	AI2746	5000	OXA-48	Hospital Universitario Marqués de Valdecilla (Santander)
177	AI2748	11	OXA-48	Hospital Universitario Marqués de Valdecilla (Santander)
178	AI2749	11	OXA-48	Hospital Universitario Marqués de Valdecilla (Santander)
179	AI2753	5000	OXA-48	Hospital Universitario Marqués de Valdecilla (Santander)
180	AI2754	5000	OXA-48	Hospital Universitario Marqués de Valdecilla (Santander)
181	AI2755	11	OXA-48	Hospital Universitario Marqués de Valdecilla (Santander)
182	AI2756	11	OXA-48	Hospital Universitario Marqués de Valdecilla (Santander)
183	AI2757	5000	OXA-48	Hospital Universitario Marqués de Valdecilla (Santander)
184	AI3063	5000	OXA-48	Hospital Universitario Marqués de Valdecilla (Santander)
185	AI2761	5000	OXA-48	Hospital Universitario Marqués de Valdecilla (Santander)
186	AI3064	307	OXA-48	Clínica Universitaria de Navarra (Navarra)
187	AI2763	147	OXA-48	Hospital Univeristario de Bellvitge (Barcelona)
188	AI2767	147	OXA-48	Hospital Univeristario de Bellvitge (Barcelona)
189	AI2768	147	OXA-48	Hospital Univeristario de Bellvitge (Barcelona)
190	AI2769	147	OXA-48	Hospital Univeristario de Bellvitge (Barcelona)
191	AI2770	147	OXA-48	Hospital Univeristario de Bellvitge (Barcelona)
192	AI3065	147	OXA-48	Hospital Univeristario de Bellvitge (Barcelona)
193	AI2771	101	OXA-48	Hospital Univeristario de Bellvitge (Barcelona)
194	AI2996	147	OXA-48	Hospital Univeristario de Bellvitge (Barcelona)
195	AI2998	147	OXA-48	Hospital Univeristario de Bellvitge (Barcelona)
196	AI2775	147	OXA-48	Hospital Univeristario de Bellvitge (Barcelona)
197	AI3001	307	OXA-48	Hospital Universitario Valencia Tres cruces (Valencia)
198	AI2776	11	OXA-48	Hospital Universitario Valencia Tres cruces (Valencia)
199	AI2779	437	NDM-23	Hospital Universitario Valencia Tres cruces (Valencia)
200	AI3002	307	OXA-48	Hospital Universitario Valencia Tres cruces (Valencia)
201	AI2782	11	OXA-48	Hospital Universitario Valencia Tres cruces (Valencia)
202	AI3067	15	OXA-48	Hospital Universitario Valencia Tres cruces (Valencia)
203	AI3003	307	OXA-48	Hospital Universitario Valencia Tres cruces (Valencia)
204	AI2784	11	OXA-48	Hospital Universitario Valencia Tres cruces (Valencia)
205	AI3004	307	OXA-48	Hospital Universitario Valencia Tres cruces (Valencia)
206	AI2785	11	OXA-48	Hospital Universitario Valencia Tres cruces (Valencia)

207	AI3006	307	OXA-48	Hospital Universitario Valencia Tres cruces (Valencia)
208	AI2787	307	OXA-48	Hospital Universitario Valencia Tres cruces (Valencia)
209	AI3007	101	NDM-1	Hospital Universitario Valencia Tres cruces (Valencia)
210	AI2786	307	OXA-48	Hospital Universitario Valencia Tres cruces (Valencia)
211	AI3069	307	OXA-48	Hospital Universitario Valencia Tres cruces (Valencia)
212	AI2789	307	OXA-48	Hospital Universitario Valencia Tres cruces (Valencia)
213	AI2791	307	OXA-48	Hospital Universitario Valencia Tres cruces (Valencia)
214	AI3009	307	OXA-48	Hospital Universitario Valencia Tres cruces (Valencia)
215	AI2792	437	OXA-48	Hospital Universitario Valencia Tres cruces (Valencia)
216	AI2793	437	NDM	Hospital Universitario Valencia Tres cruces (Valencia)
217	AI2794	307	OXA-48	Hospital Universitario Valencia Tres cruces (Valencia)
218	AI3011	13	OXA-48	Hospital Vall d'Hebron (Barcelona)
219	AI2801	101	OXA-48	Hospital Vall d'Hebron (Barcelona)
220	AI2803	628	OXA-48	Hospital Vall d'Hebron (Barcelona)
221	AI2805	512	KPC-3	Hospital Reina Sofía (Córdoba)
222	AI2806	512	KPC-3	Hospital Reina Sofía (Córdoba)
223	AI2807	512	KPC	Hospital Reina Sofía (Córdoba)
224	AI3070	15	OXA-48	Hospital Reina Sofía (Córdoba)
225	AI2808	512	KPC-3	Hospital Reina Sofía (Córdoba)
226	AI2812	512	KPC-3	Hospital Reina Sofía (Córdoba)
227	AI2814	512	KPC-3	Hospital Reina Sofía (Córdoba)
228	AI2815	512	KPC-3	Hospital Reina Sofía (Córdoba)
229	AI3071	15	OXA-48	Hospital Reina Sofía (Córdoba)
230	AI2816	512	KPC-3	Hospital Reina Sofía (Córdoba)
231	AI2817	512	KPC-3	Hospital Reina Sofía (Córdoba)
232	AN2342	147	OXA-48	Complejo Hospitalario Universitario de A Coruña (A Coruña)
233	AN2356	15	OXA-48	Hospital Universitario Lucus Augusti (Lugo)
234	-	273	KPC-2	U*
235	-	11	KPC-2	U*
236	-	101	KPC-2	U*
237	-	512	KPC-3	U*
238	-	512	KPC-3	U*
239	-	512	KPC-3	U*
240	-	512	KPC-3	U*
241	-	101	KPC-2	U*

242	-	11	KPC-2	U*
243	-	11	KPC-2	U*
244	-	15	OXA-48	U*
245	-	15	OXA-48	U*
246	-	405	OXA-48	U*
247	-	-	NCPK	Complejo Hospitalario Universitario de A Coruña (A Coruña)
248	-	-	NCPK	Complejo Hospitalario Universitario de A Coruña (A Coruña)
249	-	-	NCPK	Complejo Hospitalario Universitario de A Coruña (A Coruña)
250	-	-	NCPK	Complejo Hospitalario Universitario de A Coruña (A Coruña)
251	-	-	NCPK	Complejo Hospitalario Universitario de A Coruña (A Coruña)
252	-	-	NCPK	Complejo Hospitalario Universitario de A Coruña (A Coruña)
253	-	-	NCPK	Complejo Hospitalario Universitario de A Coruña (A Coruña)
254	-	-	NCPK	Complejo Hospitalario Universitario de A Coruña (A Coruña)
255	-	-	NCPK	Complejo Hospitalario Universitario de A Coruña (A Coruña)
256	-	-	NCPK	Complejo Hospitalario Universitario de A Coruña (A Coruña)
257	-	-	NCPK	Complejo Hospitalario Universitario de A Coruña (A Coruña)
258	-	-	NCPK	Complejo Hospitalario Universitario de A Coruña (A Coruña)
259	-	-	NCPK	Complejo Hospitalario Universitario de A Coruña (A Coruña)
260	-	-	NCPK	Complejo Hospitalario Universitario de A Coruña (A Coruña)
261	-	-	NCPK	Complejo Hospitalario Universitario de A Coruña (A Coruña)
262	-	-	NCPK	Complejo Hospitalario Universitario de A Coruña (A Coruña)
263	-	-	NCPK	Complejo Hospitalario Universitario de A Coruña (A Coruña)
264	-	-	NCPK	Complejo Hospitalario Universitario de A Coruña (A Coruña)
265	-	-	NCPK	Complejo Hospitalario Universitario de A Coruña (A Coruña)
266	-	-	NCPK	Complejo Hospitalario Universitario de A Coruña (A Coruña)
267	-	-	NCPK	Complejo Hospitalario Universitario de A Coruña (A Coruña)
268	-	-	NCPK	Complejo Hospitalario Universitario de A Coruña (A Coruña)
269	-	-	NCPK	Complejo Hospitalario Universitario de A Coruña (A Coruña)
270	-	-	NCPK	Complejo Hospitalario Universitario de A Coruña (A Coruña)
271	-	-	NCPK	Complejo Hospitalario Universitario de A Coruña (A Coruña)
272	-	-	NCPK	Complejo Hospitalario Universitario de A Coruña (A Coruña)
273	-	-	NCPK	Complejo Hospitalario Universitario de A Coruña (A Coruña)
274	-	-	NCPK	Complejo Hospitalario Universitario de A Coruña (A Coruña)
275	-	-	NCPK	Complejo Hospitalario Universitario de A Coruña (A Coruña)
276	-	-	NCPK	Complejo Hospitalario Universitario de A Coruña (A Coruña)
277	-	-	NCPK	Complejo Hospitalario Universitario de A Coruña (A Coruña)
278	-	-	NCPK	Complejo Hospitalario Universitario de A Coruña (A Coruña)
279	-	-	NCPK	Complejo Hospitalario Universitario de A Coruña (A Coruña)
280	-	-	NCPK	Complejo Hospitalario Universitario de A Coruña (A Coruña)
281	-	-	NCPK	Complejo Hospitalario Universitario de A Coruña (A Coruña)
282	-	-	NCPK	Complejo Hospitalario Universitario de A Coruña (A Coruña)
283	-	-	NCPK	Complejo Hospitalario Universitario de A Coruña (A Coruña)
284	-	-	NCPK	Complejo Hospitalario Universitario de A Coruña (A Coruña)
285	-	-	NCPK	Complejo Hospitalario Universitario de A Coruña (A Coruña)
286	-	-	NCPK	Complejo Hospitalario Universitario de A Coruña (A Coruña)

287	-	-	NCPK	Complejo Hospitalario Universitario de A Coruña (A Coruña)
288	-	-	NCPK	Complejo Hospitalario Universitario de A Coruña (A Coruña)
289	-	-	NCPK	Complejo Hospitalario Universitario de A Coruña (A Coruña)
290	-	-	NCPK	Complejo Hospitalario Universitario de A Coruña (A Coruña)
291	-	-	NCPK	Complejo Hospitalario Universitario de A Coruña (A Coruña)
292	-	-	NCPK	Complejo Hospitalario Universitario de A Coruña (A Coruña)
293	-	-	NCPK	Complejo Hospitalario Universitario de A Coruña (A Coruña)
294	-	-	NCPK	Complejo Hospitalario Universitario de A Coruña (A Coruña)
295	-	-	NCPK	Complejo Hospitalario Universitario de A Coruña (A Coruña)
296	-	-	NCPK	Complejo Hospitalario Universitario de A Coruña (A Coruña)
297	-	-	NCPK	Complejo Hospitalario Universitario de A Coruña (A Coruña)
298	-	-	NCPK	Complejo Hospitalario Universitario de A Coruña (A Coruña)
299	-	-	NCPK	Complejo Hospitalario Universitario de A Coruña (A Coruña)
300	-	-	NCPK	Complejo Hospitalario Universitario de A Coruña (A Coruña)
301	-	-	NCPK	Complejo Hospitalario Universitario de A Coruña (A Coruña)
302	-	-	NCPK	Complejo Hospitalario Universitario de A Coruña (A Coruña)
303	-	-	NCPK	Complejo Hospitalario Universitario de A Coruña (A Coruña)
304	-	-	NCPK	Complejo Hospitalario Universitario de A Coruña (A Coruña)
305	-	-	NCPK	Complejo Hospitalario Universitario de A Coruña (A Coruña)
306	-	-	NCPK	Complejo Hospitalario Universitario de A Coruña (A Coruña)
307	-	-	NCPK	Complejo Hospitalario Universitario de A Coruña (A Coruña)
308	-	-	NCPK	Complejo Hospitalario Universitario de A Coruña (A Coruña)
309	-	-	NCPK	Complejo Hospitalario Universitario de A Coruña (A Coruña)
310	-	-	NCPK	Complejo Hospitalario Universitario de A Coruña (A Coruña)
311	-	-	NCPK	Complejo Hospitalario Universitario de A Coruña (A Coruña)
312	-	-	NCPK	Complejo Hospitalario Universitario de A Coruña (A Coruña)
313	-	-	NCPK	Complejo Hospitalario Universitario de A Coruña (A Coruña)
314	-	-	NCPK	Complejo Hospitalario Universitario de A Coruña (A Coruña)
315	-	-	NCPK	Complejo Hospitalario Universitario de A Coruña (A Coruña)
316	-	-	NCPK	Complejo Hospitalario Universitario de A Coruña (A Coruña)
317	-	-	NCPK	Complejo Hospitalario Universitario de A Coruña (A Coruña)
318	-	-	NCPK	Complejo Hospitalario Universitario de A Coruña (A Coruña)
319	-	-	NCPK	Complejo Hospitalario Universitario de A Coruña (A Coruña)
320	-	-	NCPK	Complejo Hospitalario Universitario de A Coruña (A Coruña)
321	-	-	NCPK	Complejo Hospitalario Universitario de A Coruña (A Coruña)
322	-	-	NCPK	Complejo Hospitalario Universitario de A Coruña (A Coruña)
323	-	-	NCPK	Complejo Hospitalario Universitario de A Coruña (A Coruña)
324	-	-	NCPK	Complejo Hospitalario Universitario de A Coruña (A Coruña)
325	-	-	NCPK	Complejo Hospitalario Universitario de A Coruña (A Coruña)
326	-	-	NCPK	Complejo Hospitalario Universitario de A Coruña (A Coruña)
327	-	-	NCPK	Complejo Hospitalario Universitario de A Coruña (A Coruña)
328	-	-	NCPK	Complejo Hospitalario Universitario de A Coruña (A Coruña)
329	-	-	NCPK	Complejo Hospitalario Universitario de A Coruña (A Coruña)
330	-	-	NCPK	Complejo Hospitalario Universitario de A Coruña (A Coruña)
331	-	-	NCPK	Complejo Hospitalario Universitario de A Coruña (A Coruña)
332	-	-	NCPK	Complejo Hospitalario Universitario de A Coruña (A Coruña)

333	-	-	NCPK	Complejo Hospitalario Universitario de A Coruña (A Coruña)
334	-	-	NCPK	Complejo Hospitalario Universitario de A Coruña (A Coruña)
335	-	-	NCPK	Complejo Hospitalario Universitario de A Coruña (A Coruña)
336	-	-	NCPK	Complejo Hospitalario Universitario de A Coruña (A Coruña)
337	-	-	NCPK	Complejo Hospitalario Universitario de A Coruña (A Coruña)
338	-	-	NCPK	Complejo Hospitalario Universitario de A Coruña (A Coruña)
339	-	-	NCPK	Complejo Hospitalario Universitario de A Coruña (A Coruña)
340	-	-	NCPK	Complejo Hospitalario Universitario de A Coruña (A Coruña)
341	-	-	NCPK	Complejo Hospitalario Universitario de A Coruña (A Coruña)
342	-	-	NCPK	Complejo Hospitalario Universitario de A Coruña (A Coruña)
343	-	-	NCPK	Complejo Hospitalario Universitario de A Coruña (A Coruña)
344	-	-	NCPK	Complejo Hospitalario Universitario de A Coruña (A Coruña)
345	-	-	NCPK	Complejo Hospitalario Universitario de A Coruña (A Coruña)
346	-	-	NCPK	Complejo Hospitalario Universitario de A Coruña (A Coruña)
347	-	-	NCPK	Complejo Hospitalario Universitario de A Coruña (A Coruña)
348	-	-	NCPK	Complejo Hospitalario Universitario de A Coruña (A Coruña)
349	-	-	NCPK	Complejo Hospitalario Universitario de A Coruña (A Coruña)
350	-	-	NCPK	Complejo Hospitalario Universitario de A Coruña (A Coruña)
351	-	-	NCPK	Complejo Hospitalario Universitario de A Coruña (A Coruña)
352	-	-	NCPK	Complejo Hospitalario Universitario de A Coruña (A Coruña)
353	-	-	NCPK	Complejo Hospitalario Universitario de A Coruña (A Coruña)
354	-	-	NCPK	Complejo Hospitalario Universitario de A Coruña (A Coruña)
355	-	-	NCPK	Complejo Hospitalario Universitario de A Coruña (A Coruña)
356	-	-	NCPK	Complejo Hospitalario Universitario de A Coruña (A Coruña)
357	-	-	NCPK	Complejo Hospitalario Universitario de A Coruña (A Coruña)
358	-	-	NCPK	Complejo Hospitalario Universitario de A Coruña (A Coruña)
359	-	-	NCPK	Complejo Hospitalario Universitario de A Coruña (A Coruña)
360	-	-	NCPK	Complejo Hospitalario Universitario de A Coruña (A Coruña)
361	-	-	NCPK	Complejo Hospitalario Universitario de A Coruña (A Coruña)
362	-	-	NCPK	Complejo Hospitalario Universitario de A Coruña (A Coruña)
363	-	-	NCPK	Complejo Hospitalario Universitario de A Coruña (A Coruña)
364	-	-	NCPK	Complejo Hospitalario Universitario de A Coruña (A Coruña)
365	-	-	NCPK	Complejo Hospitalario Universitario de A Coruña (A Coruña)
366	-	-	NCPK	Complejo Hospitalario Universitario de A Coruña (A Coruña)
367	-	-	NCPK	Complejo Hospitalario Universitario de A Coruña (A Coruña)
368	-	-	NCPK	Complejo Hospitalario Universitario de A Coruña (A Coruña)
369	-	-	NCPK	Complejo Hospitalario Universitario de A Coruña (A Coruña)
370	-	-	NCPK	Complejo Hospitalario Universitario de A Coruña (A Coruña)
371	-	-	NCPK	Complejo Hospitalario Universitario de A Coruña (A Coruña)
372	-	-	NCPK	Complejo Hospitalario Universitario de A Coruña (A Coruña)
373	-	-	NCPK	Complejo Hospitalario Universitario de A Coruña (A Coruña)
374	-	-	NCPK	Complejo Hospitalario Universitario de A Coruña (A Coruña)
375	-	-	NCPK	Complejo Hospitalario Universitario de A Coruña (A Coruña)

376	-	-	NCPK	Complejo Hospitalario Universitario de A Coruña (A Coruña)
377	-	-	NCPK	Complejo Hospitalario Universitario de A Coruña (A Coruña)
378	-	-	NCPK	Complejo Hospitalario Universitario de A Coruña (A Coruña)
379	-	-	NCPK	Complejo Hospitalario Universitario de A Coruña (A Coruña)
380	-	-	NCPK	Complejo Hospitalario Universitario de A Coruña (A Coruña)
381	-	-	NCPK	Complejo Hospitalario Universitario de A Coruña (A Coruña)
382	-	-	NCPK	Complejo Hospitalario Universitario de A Coruña (A Coruña)
383	-	-	NCPK	Complejo Hospitalario Universitario de A Coruña (A Coruña)
384	-	-	NCPK	Complejo Hospitalario Universitario de A Coruña (A Coruña)
385	-	-	NCPK	Complejo Hospitalario Universitario de A Coruña (A Coruña)
386	-	-	NCPK	Complejo Hospitalario Universitario de A Coruña (A Coruña)
387	-	-	NCPK	Complejo Hospitalario Universitario de A Coruña (A Coruña)
388	-	-	NCPK	Complejo Hospitalario Universitario de A Coruña (A Coruña)
389	-	-	NCPK	Complejo Hospitalario Universitario de A Coruña (A Coruña)
390	-	-	NCPK	Complejo Hospitalario Universitario de A Coruña (A Coruña)
391	-	-	NCPK	Complejo Hospitalario Universitario de A Coruña (A Coruña)
392	-	-	NCPK	Complejo Hospitalario Universitario de A Coruña (A Coruña)
393	-	-	NCPK	Complejo Hospitalario Universitario de A Coruña (A Coruña)
394	-	-	NCPK	Complejo Hospitalario Universitario de A Coruña (A Coruña)
395	-	-	NCPK	Complejo Hospitalario Universitario de A Coruña (A Coruña)
396	-	-	NCPK	Complejo Hospitalario Universitario de A Coruña (A Coruña)
397	-	-	NCPK	Complejo Hospitalario Universitario de A Coruña (A Coruña)
398	-	-	NCPK	Complejo Hospitalario Universitario de A Coruña (A Coruña)
399	-	-	NCPK	Complejo Hospitalario Universitario de A Coruña (A Coruña)
400	-	-	NCPK	Complejo Hospitalario Universitario de A Coruña (A Coruña)
401	-	-	NCPK	Complejo Hospitalario Universitario de A Coruña (A Coruña)
402	-	-	NCPK	Complejo Hospitalario Universitario de A Coruña (A Coruña)
403	-	-	NCPK	Complejo Hospitalario Universitario de A Coruña (A Coruña)
404	-	-	NCPK	Complejo Hospitalario Universitario de A Coruña (A Coruña)
405	-	-	NCPK	Complejo Hospitalario Universitario de A Coruña (A Coruña)
406	-	-	NCPK	Complejo Hospitalario Universitario de A Coruña (A Coruña)
407	-	-	NCPK	Complejo Hospitalario Universitario de A Coruña (A Coruña)
408	-	-	NCPK	Complejo Hospitalario Universitario de A Coruña (A Coruña)
409	-	-	NCPK	Complejo Hospitalario Universitario de A Coruña (A Coruña)
410	-	-	NCPK	Complejo Hospitalario Universitario de A Coruña (A Coruña)
411	-	-	NCPK	Complejo Hospitalario Universitario de A Coruña (A Coruña)
412	-	-	NCPK	Complejo Hospitalario Universitario de A Coruña (A Coruña)
413	-	-	NCPK	Complejo Hospitalario Universitario de A Coruña (A Coruña)
414	-	-	NCPK	Complejo Hospitalario Universitario de A Coruña (A Coruña)
415	-	-	NCPK	Complejo Hospitalario Universitario de A Coruña (A Coruña)
416	-	-	NCPK	Complejo Hospitalario Universitario de A Coruña (A Coruña)
417	-	-	NCPK	Complejo Hospitalario Universitario de A Coruña (A Coruña)
418	-	-	NCPK	Complejo Hospitalario Universitario de A Coruña (A Coruña)

419	-	-	NCPK	Complejo Hospitalario Universitario de A Coruña (A Coruña)
420	-	-	NCPK	Complejo Hospitalario Universitario de A Coruña (A Coruña)
421	-	-	NCPK	Complejo Hospitalario Universitario de A Coruña (A Coruña)
422	-	-	NCPK	Complejo Hospitalario Universitario de A Coruña (A Coruña)
423	-	-	NCPK	Complejo Hospitalario Universitario de A Coruña (A Coruña)
424	-	-	NCPK	Complejo Hospitalario Universitario de A Coruña (A Coruña)
425	-	-	NCPK	Complejo Hospitalario Universitario de A Coruña (A Coruña)
426	-	-	NCPK	Complejo Hospitalario Universitario de A Coruña (A Coruña)
427	-	-	NCPK	Complejo Hospitalario Universitario de A Coruña (A Coruña)
428	-	-	NCPK	Complejo Hospitalario Universitario de A Coruña (A Coruña)
429	-	-	NCPK	Complejo Hospitalario Universitario de A Coruña (A Coruña)
430	-	-	NCPK	Complejo Hospitalario Universitario de A Coruña (A Coruña)
431	-	-	NCPK	Hospital Universitario Central de Asturias (Oviedo)
432	-	-	NCPK	Hospital Universitario Central de Asturias (Oviedo)
433	-	-	NCPK	Hospital Universitario Central de Asturias (Oviedo)
434	-	-	NCPK	Hospital Universitario Central de Asturias (Oviedo)
435	-	-	NCPK	Hospital Universitario Central de Asturias (Oviedo)
436	-	-	NCPK	Hospital Universitario Central de Asturias (Oviedo)
437	-	-	NCPK	Hospital Universitario Central de Asturias (Oviedo)
438	-	-	NCPK	Hospital Universitario Central de Asturias (Oviedo)
439	-	-	NCPK	Hospital Universitario Central de Asturias (Oviedo)
440	-	-	NCPK	Hospital Universitario Central de Asturias (Oviedo)
441	-	-	NCPK	Hospital Universitario Central de Asturias (Oviedo)
442	-	-	NCPK	Hospital Universitario Central de Asturias (Oviedo)
443	-	-	NCPK	Hospital Universitario Central de Asturias (Oviedo)
444	-	-	NCPK	Hospital Universitario Central de Asturias (Oviedo)
445	-	-	NCPK	Hospital Universitario Central de Asturias (Oviedo)
446	-	-	NCPK	Hospital Universitario Central de Asturias (Oviedo)
447	-	-	NCPK	Hospital Universitario Central de Asturias (Oviedo)
448	-	-	NCPK	Hospital Universitario Central de Asturias (Oviedo)
449	-	-	NCPK	Hospital Universitario Central de Asturias (Oviedo)
450	-	-	NCPK	Hospital Universitario Central de Asturias (Oviedo)
451	-	-	NCPK	Hospital Universitario Central de Asturias (Oviedo)
452	-	-	NCPK	Hospital Universitario Central de Asturias (Oviedo)
453	-	-	NCPK	Hospital Universitario Central de Asturias (Oviedo)

454	-	-	NCPK	Hospital Universitario Central de Asturias (Oviedo)
455	-	-	NCPK	Hospital Universitario Central de Asturias (Oviedo)
456	-	-	NCPK	Hospital Universitario Central de Asturias (Oviedo)
457	-	-	NCPK	Hospital Universitario Central de Asturias (Oviedo)
458	-	-	NCPK	Hospital Universitario Central de Asturias (Oviedo)
459	-	-	NCPK	Hospital Universitario Central de Asturias (Oviedo)
460	-	-	NCPK	Hospital Universitario Gregorio Marañón (Madrid)
461	-	-	NCPK	Hospital Universitario Gregorio Marañón (Madrid)
462	-	-	NCPK	Hospital Universitario Gregorio Marañón (Madrid)
463	-	-	NCPK	Hospital Universitario Gregorio Marañón (Madrid)
464	-	-	NCPK	Hospital Universitario Gregorio Marañón (Madrid)
465	-	-	NCPK	Hospital Universitario Gregorio Marañón (Madrid)
466	-	-	NCPK	Hospital Universitario Gregorio Marañón (Madrid)
467	-	-	NCPK	Hospital Universitario Gregorio Marañón (Madrid)
468	-	-	NCPK	Hospital Universitario Gregorio Marañón (Madrid)
469	-	-	NCPK	Hospital Universitario Gregorio Marañón (Madrid)
470	-	-	NCPK	Hospital Universitario Gregorio Marañón (Madrid)
471	-	-	NCPK	Hospital Universitario Gregorio Marañón (Madrid)
472	-	-	NCPK	Hospital Universitario Gregorio Marañón (Madrid)
473	-	-	NCPK	Hospital Universitario Gregorio Marañón (Madrid)
474	-	-	NCPK	Hospital Universitario Gregorio Marañón (Madrid)
475	-	-	NCPK	Hospital Universitario Gregorio Marañón (Madrid)
476	-	-	NCPK	Hospital Universitario Gregorio Marañón (Madrid)
477	-	-	NCPK	Hospital Universitario Gregorio Marañón (Madrid)
478	-	-	NCPK	Hospital Universitario Gregorio Marañón (Madrid)
479	-	-	NCPK	Hospital Universitario Gregorio Marañón (Madrid)

b. Validation

Isolate	Sequencing code	ST	Resistance mechanism	Sample of isolation	Hospital/City where strain was isolated
1	AI2584	11	OXA-48	Urine	Hospital Universitario Gregorio Marañón (Madrid)
2	AI2591	11	OXA-48	Respiratory sample	Hospital Universitario Gregorio Marañón (Madrid)
3	AI2592	405	OXA-48	Urine	Hospital Universitario Gregorio Marañón (Madrid)
4	AI3018	15	OXA-48	Urine	Hospital Universitario Gregorio Marañón (Madrid)
5	AI2608	392	OXA-48	Urine	Hospital Universitario Gregorio Marañón (Madrid)
6	AI2610	11	OXA-48	Urine	Hospital Universitario Gregorio Marañón (Madrid)
7	AI2629	512	KPC-3	Urine	Hospital Puerta del Mar (Cádiz)
8	AI2633	11	OXA-48	Wound	Hospital Puerta del Mar (Cádiz)
9	AI2837	512	KPC-3	Wound	Hospital Puerta del Mar (Cádiz)
10	AI2637	429	OXA-48	Urine	Hospital Clinic (Barcelona)
11	AH0325	392	OXA-48	Urine	Hospital Clinic (Barcelona)
12	AI2841	392	OXA-48	Peritoneal fluid	Hospital Clinic (Barcelona)
13	AI2638	307	OXA-48	Urine	Hospital Clinic (Barcelona)
14	AI2612	15	OXA-48	Urine	Hospital Arquitecto Marcide (Ferrol)
15	AI2849	147	OXA-48	Urine	Hospital Universitario Mutua de Terrassa (Terrassa)
16	AI2854	152	OXA-48	Wound	Hospital Universitario Mutua de Terrassa (Terrassa)
17	AI2855	147	OXA-48	Urine	Hospital Universitario Mutua de Terrassa (Terrassa)
18	AI2863	307	OXA-48	Abscess	Hospital Universitario La Paz (Madrid)
19	AI3023	15	OXA-48	Respiratory sample	Hospital Universitario La Paz (Madrid)
20	AI2871	11	OXA-48	Abscess	Hospital Universitario Ramón y Cajal (Madrid)
21	AI2877	11	OXA-48	Urine	Hospital Universitario Ramón y Cajal (Madrid)
22	AI2883	307	KPC-3	Urine	Hospital Universitario Ramón y Cajal (Madrid)
23	AI2886	15	OXA-48	Urine	Hospital Universitario Ramón y Cajal (Madrid)
24	AI2646	307	KPC-3	blood culture	Hospital Universitario Ramón y Cajal (Madrid)
25	AI2889	307	KPC-3	Urine	Hospital Universitario Ramón y Cajal (Madrid)
26	AI2891	11	OXA-48	Urine	Hospital Universitario Ramón y Cajal (Madrid)
27	AI2910	11	OXA-48	Urine	Hospital Universitario La Princesa (Madrid)
28	AH0329	392	OXA-48	Urine	Hospital Universitario La Princesa (Madrid)
29	AI2920	392	OXA-48	Urine	Hospital Universitario La Princesa (Madrid)
30	AI2921	405	OXA-48	Urine	Hospital Universitario La Princesa (Madrid)
31	AI2930	307	OXA-48	Urine	Hospital Universitario La Princesa (Madrid)
32	AI2667	485	OXA-48	blood culture	Hospital Universitario Central de Asturias (Oviedo)
33	AI3031	307	OXA-48	Urine	Hospital Universitario Central de Asturias (Oviedo)

34	AI2664	147	OXA-48	Urine	Hospital Universitario Central de Asturias (Oviedo)
35	AI2665	15	OXA-48	Urine	Hospital Universitario Central de Asturias (Oviedo)
36	AI2942	326	OXA-48	blood culture	Hospital Universitario Central de Asturias (Oviedo)
37	AI3036	326	OXA-48	Wound	Hospital Universitario Central de Asturias (Oviedo)
38	AI2693	567	OXA-48	Wound	Hospital Universitario Central de Asturias (Oviedo)
39	AI2952	326	OXA-48	Urine	Hospital Universitario Central de Asturias (Oviedo)
40	AI2954	11	OXA-48	blood culture culture	Hospital de Guadalajara (Guadalajara)
41	AI2957	307	OXA-48	Urine	Hospital de Guadalajara (Guadalajara)
42	AH0331	104	OXA-48	Urine	Hospital de Guadalajara (Guadalajara)
43	AI2971	11	OXA-48	Urine	Hospital de Guadalajara (Guadalajara)
44	AI3041	307	OXA-48	Urine	Hospital de Guadalajara (Guadalajara)
45	AI3042	11	KPC-2	blood culture	Hospital Virgen de la Salud (Toledo)
46	AI3046	512	KPC-3	Urine	Lab. Dr. Echevarne
47	AI3047	11	KPC-2	Respiratory sample	Hospital General Universitario de Ciudad Real (Ciudad Real)
48	AI3051	11	KPC-2	-	U*
49	AI3052	11	KPC-2	-	U*
50	AI3055	1962	KPC-2	Urine	Hospital Virgen de la Salud (Toledo)
51	AI2723	258	KPC-3	Urine	Hospital Universitario Virgen de las Nieves (Granada)
52	AI2714	512	KPC-3	blood culture	Hospital General La Mancha Centro (Ciudad Real)
53	AN2341	5000	OXA-48	Urine	Hospital Universitario Marqués de Valdecilla (Santander)
54	AI2742	11	OXA-48	Biopsy	Hospital Universitario Marqués de Valdecilla (Santander)
55	AI2750	11	OXA-48	Urine	Hospital Universitario Marqués de Valdecilla (Santander)
56	AI2751	5000	OXA-48	Wound	Hospital Universitario Marqués de Valdecilla (Santander)
57	AI3062	5000	OXA-48	Wound	Hospital Universitario Marqués de Valdecilla (Santander)
58	AI2764	147	OXA-48	Respiratory sample	Hospital Univeristario de Bellvitge (Barcelona)
59	AI2765	147	OXA-48	Urine	Hospital Univeristario de Bellvitge (Barcelona)
60	AI2766	147	OXA-48	Urine	Hospital Univeristario de Bellvitge (Barcelona)
61	AI2993	147	OXA-48	Wound	Hospital Univeristario de Bellvitge (Barcelona)
62	AI2773	307	OXA-48	Urine	Hospital Univeristario de Bellvitge (Barcelona)
63	AI2777	307	OXA-48	Urine	Hospital Universitario Valencia Tres cruces (Valencia)
64	AI2778	11	OXA-48	Urine	Hospital Universitario Valencia Tres cruces (Valencia)
65	AI2780	11	OXA-48	Respiratory sample	Hospital Universitario Valencia Tres cruces (Valencia)
66	AI2781	307	OXA-48	Urine	Hospital Universitario Valencia Tres cruces (Valencia)
67	AI2790	11	OXA-48	Bile	Hospital Universitario Valencia Tres cruces (Valencia)
68	AI2800	405	OXA-48	Bile	Hospital Vall d'Hebron (Barcelona)

69	AI2809	512	KPC-3	Peritoneal fluid	Hospital Reina Sofía (Córdoba)
70	AI2810	512	KPC-3	Wound	Hospital Reina Sofía (Córdoba)
71	AI2813	512	KPC-3	Abscess	Hospital Reina Sofía (Córdoba)
72	AI2811	512	KPC-3	blood culture	Hospital Reina Sofía (Córdoba)
73	AN2344	15	OXA-48	Wound	Complejo Hospitalario Universitario de A Coruña (A Coruña)
74	AN2357	15	OXA-48	Urine	Hospital Universitario Lucus Augusti (Lugo)
75	-	11	KPC-2	-	U*
76	-	101	KPC-2	-	U*
77	-	512	KPC-3	-	U*
78	-	11	OXA-48	-	U*
79	-	-	NCPK	Urine	Complejo Hospitalario Universitario de A Coruña (A Coruña)
80	-	-	NCPK	Urine	Complejo Hospitalario Universitario de A Coruña (A Coruña)
81	-	-	NCPK	Urine	Complejo Hospitalario Universitario de A Coruña (A Coruña)
82	-	-	NCPK	Urine	Complejo Hospitalario Universitario de A Coruña (A Coruña)
83	-	-	NCPK	Urine	Complejo Hospitalario Universitario de A Coruña (A Coruña)
84	-	-	NCPK	Respiratory sample	Complejo Hospitalario Universitario de A Coruña (A Coruña)
85	-	-	NCPK	Urine	Complejo Hospitalario Universitario de A Coruña (A Coruña)
86	-	-	NCPK	Urine	Complejo Hospitalario Universitario de A Coruña (A Coruña)
87	-	-	NCPK	Urine	Complejo Hospitalario Universitario de A Coruña (A Coruña)
88	-	-	NCPK	Urine	Complejo Hospitalario Universitario de A Coruña (A Coruña)
89	-	-	NCPK	Wound	Complejo Hospitalario Universitario de A Coruña (A Coruña)
90	-	-	NCPK	Urine	Complejo Hospitalario Universitario de A Coruña (A Coruña)
91	-	-	NCPK	Urine	Complejo Hospitalario Universitario de A Coruña (A Coruña)
92	-	-	NCPK	Urine	Complejo Hospitalario Universitario de A Coruña (A Coruña)
93	-	-	NCPK	Urine	Complejo Hospitalario Universitario de A Coruña (A Coruña)
94	-	-	NCPK	Urine	Complejo Hospitalario Universitario de A Coruña (A Coruña)
95	-	-	NCPK	Urine	Complejo Hospitalario Universitario de A Coruña (A Coruña)
96	-	-	NCPK	Urine	Complejo Hospitalario Universitario de A Coruña (A Coruña)
97	-	-	NCPK	Urine	Complejo Hospitalario Universitario de A Coruña (A Coruña)
98	-	-	NCPK	Urine	Complejo Hospitalario Universitario de A Coruña (A Coruña)
99	-	-	NCPK	Urine	Complejo Hospitalario Universitario de A Coruña (A Coruña)
100	-	-	NCPK	Urine	Complejo Hospitalario Universitario de A Coruña (A Coruña)
101	-	-	NCPK	Urine	Complejo Hospitalario Universitario de A Coruña (A Coruña)
102	-	-	NCPK	Respiratory sample	Complejo Hospitalario Universitario de A Coruña (A Coruña)
103	-	-	NCPK	Urine	Complejo Hospitalario Universitario de A Coruña (A Coruña)
104	-	-	NCPK	Urine	Complejo Hospitalario Universitario de A Coruña (A Coruña)
105	-	-	NCPK	Respiratory sample	Complejo Hospitalario Universitario de A Coruña (A Coruña)
106	-	-	NCPK	Urine	Complejo Hospitalario Universitario de A Coruña (A Coruña)
107	-	-	NCPK	Urine	Complejo Hospitalario Universitario de A Coruña (A Coruña)
108	-	-	NCPK	Urine	Complejo Hospitalario Universitario de A Coruña (A Coruña)
109	-	-	NCPK	Urine	Complejo Hospitalario Universitario de A Coruña (A Coruña)

110	-	-	NCPK	Urine	Complejo Hospitalario Universitario de A Coruña (A Coruña)
111	-	-	NCPK	Urine	Complejo Hospitalario Universitario de A Coruña (A Coruña)
112	-	-	NCPK	Urine	Complejo Hospitalario Universitario de A Coruña (A Coruña)
113	-	-	NCPK	Urine	Complejo Hospitalario Universitario de A Coruña (A Coruña)
114	-	-	NCPK	Urine	Complejo Hospitalario Universitario de A Coruña (A Coruña)
115	-	-	NCPK	Urine	Complejo Hospitalario Universitario de A Coruña (A Coruña)
116	-	-	NCPK	Urine	Complejo Hospitalario Universitario de A Coruña (A Coruña)
117	-	-	NCPK	Urine	Complejo Hospitalario Universitario de A Coruña (A Coruña)
118	-	-	NCPK	Urine	Complejo Hospitalario Universitario de A Coruña (A Coruña)
119	-	-	NCPK	Urine	Complejo Hospitalario Universitario de A Coruña (A Coruña)
120	-	-	NCPK	Urine	Complejo Hospitalario Universitario de A Coruña (A Coruña)
121	-	-	NCPK	Urine	Complejo Hospitalario Universitario de A Coruña (A Coruña)
122	-	-	NCPK	Urine	Complejo Hospitalario Universitario de A Coruña (A Coruña)
123	-	-	NCPK	Urine	Complejo Hospitalario Universitario de A Coruña (A Coruña)
124	-	-	NCPK	Urine	Complejo Hospitalario Universitario de A Coruña (A Coruña)
125	-	-	NCPK	Urine	Complejo Hospitalario Universitario de A Coruña (A Coruña)
126	-	-	NCPK	Urine	Complejo Hospitalario Universitario de A Coruña (A Coruña)
127	-	-	NCPK	Urine	Complejo Hospitalario Universitario de A Coruña (A Coruña)
128	-	-	NCPK	Urine	Complejo Hospitalario Universitario de A Coruña (A Coruña)
129	-	-	NCPK	Urine	Complejo Hospitalario Universitario de A Coruña (A Coruña)
130	-	-	NCPK	Urine	Complejo Hospitalario Universitario de A Coruña (A Coruña)
131	-	-	NCPK	Urine	Complejo Hospitalario Universitario de A Coruña (A Coruña)
132	-	-	NCPK	blood culture	Complejo Hospitalario Universitario de A Coruña (A Coruña)
133	-	-	NCPK	Urine	Complejo Hospitalario Universitario de A Coruña (A Coruña)
134	-	-	NCPK	Urine	Complejo Hospitalario Universitario de A Coruña (A Coruña)
135	-	-	NCPK	blood culture	Complejo Hospitalario Universitario de A Coruña (A Coruña)
136	-	-	NCPK	Urine	Complejo Hospitalario Universitario de A Coruña (A Coruña)
137	-	-	NCPK	Urine	Complejo Hospitalario Universitario de A Coruña (A Coruña)
138	-	-	NCPK	Urine	Complejo Hospitalario Universitario de A Coruña (A Coruña)
139	-	-	NCPK	Urine	Complejo Hospitalario Universitario de A Coruña (A Coruña)
140	-	-	NCPK	Urine	Complejo Hospitalario Universitario de A Coruña (A Coruña)
141	-	-	NCPK	Urine	Complejo Hospitalario Universitario de A Coruña (A Coruña)
142	-	-	NCPK	Urine	Complejo Hospitalario Universitario de A Coruña (A Coruña)
143	-	-	NCPK	Urine	Complejo Hospitalario Universitario de A Coruña (A Coruña)
144	-	-	NCPK	Urine	Complejo Hospitalario Universitario de A Coruña (A Coruña)
145	-	-	NCPK	Urine	Complejo Hospitalario Universitario de A Coruña (A Coruña)
146	-	-	NCPK	Urine	Complejo Hospitalario Universitario de A Coruña (A Coruña)
147	-	-	NCPK	Respiratory sample	Complejo Hospitalario Universitario de A Coruña (A Coruña)
148	-	-	NCPK	Urine	Complejo Hospitalario Universitario de A Coruña (A Coruña)
149	-	-	NCPK	Urine	Complejo Hospitalario Universitario de A Coruña (A Coruña)
150	-	-	NCPK	Urine	Complejo Hospitalario Universitario de A Coruña (A Coruña)
151	-	-	NCPK	Urine	Complejo Hospitalario Universitario de A Coruña (A Coruña)
152	-	-	NCPK	Urine	Complejo Hospitalario Universitario de A Coruña (A Coruña)

153	-	-	NCPK	Urine	Complejo Hospitalario Universitario de A Coruña (A Coruña)
154	-	-	NCPK	Urine	Complejo Hospitalario Universitario de A Coruña (A Coruña)
155	-	-	NCPK	Wound	Complejo Hospitalario Universitario de A Coruña (A Coruña)
156	-	-	NCPK	Urine	Complejo Hospitalario Universitario de A Coruña (A Coruña)
157	-	-	NCPK	Urine	Complejo Hospitalario Universitario de A Coruña (A Coruña)
158	-	-	NCPK	Urine	Complejo Hospitalario Universitario de A Coruña (A Coruña)
159	-	-	NCPK	Urine	Complejo Hospitalario Universitario de A Coruña (A Coruña)
160	-	-	NCPK	Respiratory sample	Complejo Hospitalario Universitario de A Coruña (A Coruña)
161	-	-	NCPK	Urine	Complejo Hospitalario Universitario de A Coruña (A Coruña)
162	-	-	NCPK	Urine	Complejo Hospitalario Universitario de A Coruña (A Coruña)
163	-	-	NCPK	Urine	Complejo Hospitalario Universitario de A Coruña (A Coruña)
164	-	-	NCPK	Urine	Complejo Hospitalario Universitario de A Coruña (A Coruña)
165	-	-	NCPK	Urine	Complejo Hospitalario Universitario de A Coruña (A Coruña)
166	-	-	NCPK	Urine	Complejo Hospitalario Universitario de A Coruña (A Coruña)
167	-	-	NCPK	Urine	Complejo Hospitalario Universitario de A Coruña (A Coruña)
168	-	-	NCPK	Urine	Complejo Hospitalario Universitario de A Coruña (A Coruña)
169	-	-	NCPK	Urine	Complejo Hospitalario Universitario de A Coruña (A Coruña)
170	-	-	NCPK	Urine	Complejo Hospitalario Universitario de A Coruña (A Coruña)
171	-	-	NCPK	Urine	Complejo Hospitalario Universitario de A Coruña (A Coruña)
172	-	-	NCPK	Urine	Complejo Hospitalario Universitario de A Coruña (A Coruña)
173	-	-	NCPK	Urine	Complejo Hospitalario Universitario de A Coruña (A Coruña)
174	-	-	NCPK	Urine	Complejo Hospitalario Universitario de A Coruña (A Coruña)
175	-	-	NCPK	Urine	Complejo Hospitalario Universitario de A Coruña (A Coruña)
176	-	-	NCPK	Urine	Complejo Hospitalario Universitario de A Coruña (A Coruña)
177	-	-	NCPK	Urine	Complejo Hospitalario Universitario de A Coruña (A Coruña)
178	-	-	NCPK	Urine	Complejo Hospitalario Universitario de A Coruña (A Coruña)
179	-	-	NCPK	Urine	Complejo Hospitalario Universitario de A Coruña (A Coruña)
180	-	-	NCPK	Urine	Complejo Hospitalario Universitario de A Coruña (A Coruña)
181	-	-	NCPK	Urine	Complejo Hospitalario Universitario de A Coruña (A Coruña)
182	-	-	NCPK	Urine	Complejo Hospitalario Universitario de A Coruña (A Coruña)
183	-	-	NCPK	Urine	Complejo Hospitalario Universitario de A Coruña (A Coruña)
184	-	-	NCPK	Urine	Complejo Hospitalario Universitario de A Coruña (A Coruña)
185	-	-	NCPK	Urine	Complejo Hospitalario Universitario de A Coruña (A Coruña)
186	-	-	NCPK	Urine	Complejo Hospitalario Universitario de A Coruña (A Coruña)
187	-	-	NCPK	Urine	Complejo Hospitalario Universitario de A Coruña (A Coruña)
188	-	-	NCPK	Urine	Complejo Hospitalario Universitario de A Coruña (A Coruña)
189	-	-	NCPK	Urine	Complejo Hospitalario Universitario de A Coruña (A Coruña)
190	-	-	NCPK	Urine	Complejo Hospitalario Universitario de A Coruña (A Coruña)
191	-	-	NCPK	Urine	Complejo Hospitalario Universitario de A Coruña (A Coruña)
192	-	-	NCPK	Urine	Complejo Hospitalario Universitario de A Coruña (A Coruña)
193	-	-	NCPK	Urine	Complejo Hospitalario Universitario de A Coruña (A Coruña)
194	-	-	NCPK	Urine	Complejo Hospitalario Universitario de A Coruña (A Coruña)
195	-	-	NCPK	Urine	Complejo Hospitalario Universitario de A Coruña (A Coruña)

196	-	-	NCPK	Urine	Complejo Hospitalario Universitario de A Coruña (A Coruña)
197	-	-	NCPK	Urine	Complejo Hospitalario Universitario de A Coruña (A Coruña)
198	-	-	NCPK	Urine	Complejo Hospitalario Universitario de A Coruña (A Coruña)
199	-	-	NCPK	Urine	Complejo Hospitalario Universitario de A Coruña (A Coruña)
200	-	-	NCPK	Urine	Complejo Hospitalario Universitario de A Coruña (A Coruña)
201	-	-	NCPK	Urine	Complejo Hospitalario Universitario de A Coruña (A Coruña)
202	-	-	NCPK	Urine	Complejo Hospitalario Universitario de A Coruña (A Coruña)
203	-	-	NCPK	Urine	Complejo Hospitalario Universitario de A Coruña (A Coruña)
204	-	-	NCPK	Urine	Complejo Hospitalario Universitario de A Coruña (A Coruña)
205	-	-	NCPK	Urine	Hospital Universitario Central de Asturias (Oviedo)
206	-	-	NCPK	Urine	Hospital Universitario Central de Asturias (Oviedo)
207	-	-	NCPK	Urine	Hospital Universitario Central de Asturias (Oviedo)
208	-	-	NCPK	Urine	Hospital Universitario Central de Asturias (Oviedo)
209	-	-	NCPK	Urine	Hospital Universitario Central de Asturias (Oviedo)
210	-	-	NCPK	Urine	Hospital Universitario Central de Asturias (Oviedo)
211	-	-	NCPK	Urine	Hospital Universitario Central de Asturias (Oviedo)
212	-	-	NCPK	Wound	Hospital Universitario Central de Asturias (Oviedo)
213	-	-	NCPK	Urine	Hospital Universitario Central de Asturias (Oviedo)
214	-	-	NCPK	Urine	Hospital Universitario Central de Asturias (Oviedo)
215	-	-	NCPK	Urine	Hospital Universitario Central de Asturias (Oviedo)
216	-	-	NCPK	Wound	Hospital Universitario Central de Asturias (Oviedo)
217	-	-	NCPK	Wound	Hospital Universitario Central de Asturias (Oviedo)
218	-	-	NCPK	blood culture	Hospital Universitario Gregorio Marañón (Madrid)
219	-	-	NCPK	urine	Hospital Universitario Gregorio Marañón (Madrid)
220	-	-	NCPK	respiratory sample	Hospital Universitario Gregorio Marañón (Madrid)
221	-	-	NCPK	catheter	Hospital Universitario Gregorio Marañón (Madrid)
222	-	-	NCPK	respiratory sample	Hospital Universitario Gregorio Marañón (Madrid)
223	-	-	NCPK	blood culture	Hospital Universitario Gregorio Marañón (Madrid)
224	-	-	NCPK	blood culture	Hospital Universitario Gregorio Marañón (Madrid)
225	-	-	NCPK	blood culture	Hospital Universitario Gregorio Marañón (Madrid)
226	-	-	NCPK	blood culture	Hospital Universitario Gregorio Marañón (Madrid)
227	-	-	NCPK	abscess	Hospital Universitario Gregorio Marañón (Madrid)
228	-	-	NCPK	respiratory sample	Hospital Universitario Gregorio Marañón (Madrid)
229	-	-	NCPK	blood culture	Hospital Universitario Gregorio Marañón (Madrid)
230	-	-	NCPK	blood culture	Hospital Universitario Gregorio Marañón (Madrid)
231	-	-	NCPK	urine	Hospital Universitario Gregorio Marañón (Madrid)
232	-	-	NCPK	wound	Hospital Universitario Gregorio Marañón (Madrid)
233	-	-	NCPK	blood culture	Hospital Universitario Gregorio Marañón (Madrid)
234	-	-	NCPK	blood culture	Hospital Universitario Gregorio Marañón (Madrid)
235	-	-	NCPK	blood culture	Hospital Universitario Gregorio Marañón (Madrid)
236	-	-	NCPK	wound	Hospital Universitario Gregorio Marañón (Madrid)

*U: unknown origin

Table 2. MALDI-TOF MS based CPK optimized hyperparameters for: a) CPK identification and b) differentiation of the carbapenemase type.

a)

Method	Algorithm**	C[1]	n Estimators[2]	Min simples leaf[3]	Min simples split[4]	n Neighbors[5]
M_{LINEAR}	PCA-SVM	0.01	-	-	-	
	SVM	0.001	-	-	-	--
	RF	-	600	1	2	-
	NCA-KNN	-	-	-	-	8
	KNN	-	-	-	-	2
$M_{THRESHOLD}$	PCA-SVM*	0.001	-	-	-	-
	SVM	1	-	-	-	-
	RF	-	600	1	2	-
	NCA-KNN	-	-	-	-	2
	KNN	-	-	-	-	5
$M_{LINEAR-3K}$	PCA-SVM	0.1	-	-	-	-
	SVM	0.001	-	-	-	-
	RF	-	200	1	2	-
	NCA-KNN	-	-	-	-	9
	KNN	-	-	-	-	8
$M_{THRESHOLD-3K}$	PCA-SVM*	0.1	-	-	-	-
	SVM	0.001	-	-	-	-
	RF	-	200	1	2	-
	NCA-KNN	-	-	-	-	8
	KNN	-	-	-	-	9

b)

Method	Algorithm	C	n Estimators	Min simples leaf	Min simples split	n Neighbors
M_{LINEAR}	SVM	10		--	-	-
	RF	-	100	1	2	-
	NCA-KNN	-	--		-	6
	KNN	-	-	-	-	3
$M_{THRESHOLD}$	SVM	10	-	-	-	-
	RF	-	600	1	2	-
	NCA-KNN	-		-	-	6

	**KNN**	-	-	-	-	3
**$M_{LINEAR\text{-}3K}$**	**SVM**	10	-	-	-	-
	**RF**	-	200	1	2	
	**NCA-KNN**	-	-	-	-	3
	**KNN**	-	-	-	-	3
**$M_{THRESHOLD\text{-}3k}$**	**SVM**	0.001	-	-	-	-
	**RF**	-	600	1	2	
	**NCA-KNN**	-	-	-	-	6
	**KNN**	-	-	-	-	3

[1] C Regularization parameter of SVM. The strength of the regularization is inversely proportional to C, must be strictly positive.

[2] n Estimators, the number of trees in the forest

[3] Min simples leaf, the minimum number of samples required to be at a leaf node

[4] Min simples split, the minimum number of samples required to split an internal node all for RF

[5] n Neighbors, number of neighbors to use for KNN.

*Filter peaks mode on: Only peaks that appear in at least 80% of the spectra of each category are used for the analysis.

**Note that PLS does not use hyperparameters.

Table 3. MALDI-TOF MS-based CPK prediction with detailed metrics for all method-analytical algorithm combinations. All values are percentages.

a.1)

Method	Algorithm	Accuracy	F1 Score	Sensitivity	Specificity
M_{LINEAR}	PLS	99.37	99.39	100	98.71
	PCA-SVM	95.37	95.47	95.87	94.85
	SVM	99.58	99.59	100	99.14
	RF	99.79	99.79	100	99.57
	NCA-KNN	97.05	97.15	98.76	95.28
	KNN	95.79	96.03	100	91.42
$M_{THRESHOLD}$	PLS	98.95	98.98	99.59	98.28
	PCA-SVM	87.58	87.63	86.36	88.84
	SVM	99.16	99.18	99.59	98.71
	RF	99.37	99.38	99.59	99.14
	NCA-KNN	98.95	98,96	98.76	99.14
	KNN	97.26	97.35	98.76	95.71
$M_{LINEAR\text{-}3K}$	PLS	99.37	99.39	100	98.71
	PCA-SVM	95.58	95.69	96.28	94.85
	SVM	99.37	99.39	100	98.71
	RF	99.79	99.79	99.59	100
	NCA-KNN	97.89	97.97	99.59	96.14
	KNN	96.42	96.61	100	92.7
$M_{THRESHOLD\text{-}3K}$	PLS	98.11	98.17	99.59	96.57
	PCA-SVM	89.68	90.06	91.74	87.55
	SVM	98.53	98.57	99.59	97.42
	RF	98.11	98.13	97.93	98.28
	NCA-KNN	97.26	97.32	97.52	97
	KNN	94.32	94.46	95.04	93.56

a.2)

Method	Algorithm	Accuracy
M_{LINEAR}	PLS	87.6
	SVM	89.26
	RF	90.91
	NCA-KNN	87.19
	KNN	85.12
$M_{THRESHOLD}$	PLS	86.36
	SVM	86.36
	RF	90.08
	NCA-KNN	84.71

	KNN	83.88
**M$_{LINEAR-3K}$**	PLS	85.54
	SVM	89.67
	RF	90.91
	NCA-KNN	88.84
	KNN	83.47
**M$_{THRESHOLD-3k}$**	PLS	88.84
	SVM	85.54
	RF	89.67
	NCA-KNN	86.36
	KNN	84.71

b.1)

Method	Algorithm	Accuracy	F1 Score	Sensitivity	Specificity
**M$_{LINEAR}$**	**PLS**	96.09	94.48	100	94.12
	**PCA-SVM**	90.43	87.36	98.7	86.27
	**SVM**	97.39	96.25	100	96.08
	**RF**	97.83	96.85	100	96.73
	**NCA-KNN**	90	87.01	100	84.97
	**KNN**	91.3	88.5	100	86.93
**M$_{THRESHOLD}$**	**PLS**	88.7	85.56	100	83.01
	**PCA-SVM**	74.78	70.71	90.91	66.67
	**SVM**	97.83	96.85	100	96.73
	**RF**	95.65	93.9	100	93.46
	**NCA-KNN**	82.17	78.3	96.1	75.16
	**KNN**	82.61	78.95	97.4	75.16
**M$_{LINEAR-3K}$**	**PLS**	95.22	93.33	100	92.81
	**PCA-SVM**	91.3	88.5	100	86.93
	**SVM**	96.52	95.06	100	94.77
	**RF**	96.96	95.65	100	95.42
	**NCA-KNN**	91.3	88.5	100	86.93
	**KNN**	90	87.01	100	84.97
**M$_{THRESHOLD-3K}$**	**PLS**	85.22	81.92	100	77.78
	**PCA-SVM**	75.65	73.08	98.7	64.05
	**SVM**	88.26	85.08	100	82.35
	**RF**	85.65	82.35	100	78.43
	**NCA-KNN**	78.7	75.86	100	67.97
	**KNN**	81.3	75.98	88.31	77.78

b.2)

Method	Algorithm	Accuracy
M_{LINEAR}	PLS	85.71
	SVM	93.51
	RF	98.7
	NCA-KNN	96.1
	KNN	85.71
$M_{THRESHOLD}$	PLS	76.62
	SVM	84.42
	RF	94.81
	NCA-KNN	87.01
	KNN	84.42
$M_{LINEAR-3K}$	PLS	84.42
	SVM	94.81
	RF	92.66
	NCA-KNN	97.4
	KNN	83.12
$M_{THRESHOLD-3k}$	PLS	85.71
	SVM	88.31
	RF	94.81
	NCA-KNN	85.71
	KNN	83.12

Results of the prediction performance and quality metrics for evaluation of all tested combinations of methods-analytical algorithms in each scenario a) training set and b) validation set, for 1) CPK prediction and 2) identification of the carbapenemase type. All values represent percentages.

Table 4. Metrics detailed information for all methods-algorithms of analysis combinations regarding the AUROC and AUPRC.

a) Identification of CPK

		AUROC	AUPRC	AP
M_{FULL}	**PLS**	0,988	0,96	0,95
	**SVM**	0,983	0,89	0,9
	**PCA-SVM**	0,962	0,85	0,86
	**RF**	1	1	1
$M_{FULL-3K}$	**PLS**	0,989	0,96	0,95
	**SVM**	0,986	0,9	0,91
	**PCA-SVM**	0,963	0,88	0,88
	**RF**	1	1	1

a) Identification of the type of carbapenemase

OXA-48					KPC				
		AUROC	AUPRC	AP			AUROC	AUPRC	AP
M_{FULL}	**PLS**	0,948	0,98	0,98	M_{FULL}	**PLS**	0,948	0,89	0,89
	**SVM**	0,967	0,99	0,99		**SVM**	0,964	0,95	0,95
	**RF**	0,978	0,99	0,99		**RF**	0,975	0,97	0,97
$M_{FULL-3K}$	**PLS**	0,95	0,98	0,98	$M_{FULL-3K}$	**PLS**	0,95	0,98	0,98
	**SVM**	0,965	0,98	0,99		**SVM**	0,961	0,95	0,95
	**RF**	0,979	0,99	0,99		**RF**	0,982	0,97	0,98

Figure 1. Receiver operating characteristic curves values for the validation stage by area under curve (AUC) and precision–recall curves values obtained by AUC and average precision (AP) methods of best-performance for CPK prediction after applying the RF algorithm.

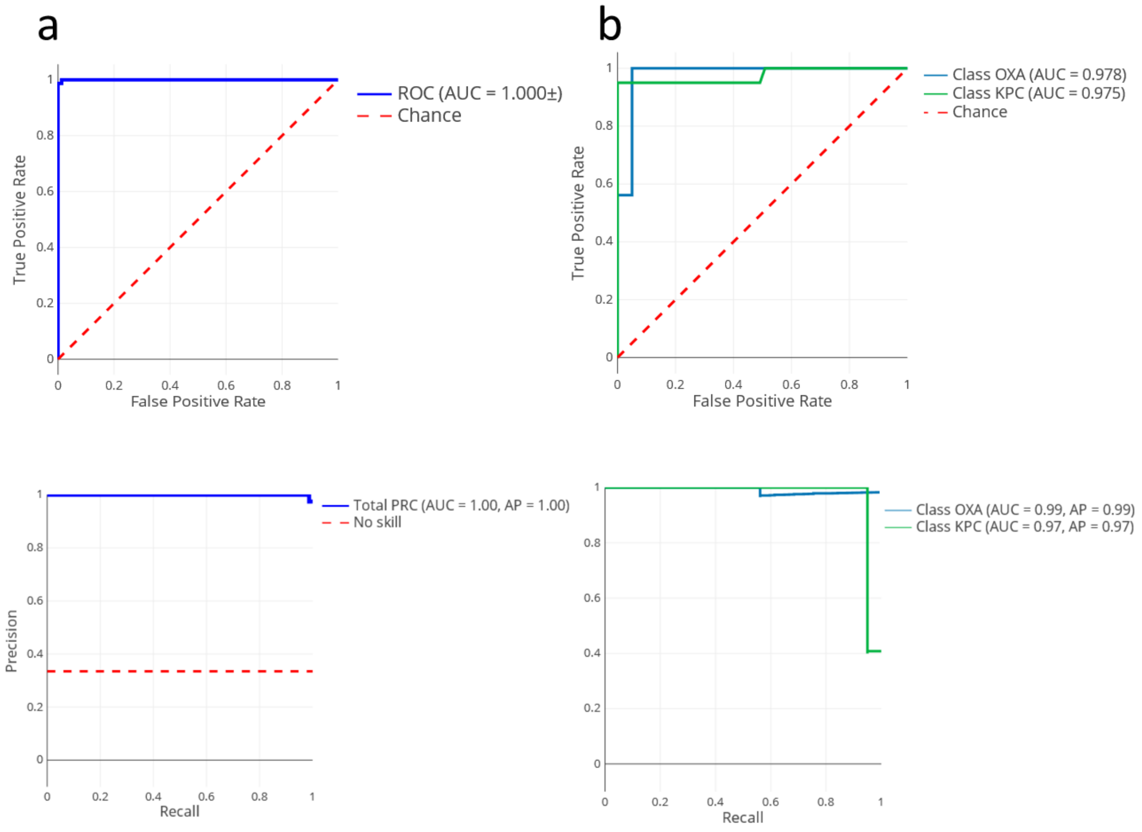

a) AUROC and AUPRC for CPK prediction applying the RF algorithm and the M_{LINEAR} method.

b) AUROC and AUPRC for differentiation of the carbapenemase type after applying the RF algorithm and the M_{LINEAR} method.
